# Fundamental aspects of sucrose metabolism reveal a trophic link between *Rhodospirillum rubrum* and *Rhodobacter capsulatus*

**DOI:** 10.1128/mbio.03717-25

**Published:** 2026-02-13

**Authors:** Manon Gilson, Guillaume Bayon-Vicente, Simone Krings, Laura Toubeau, Ruddy Wattiez, Baptiste Leroy

**Affiliations:** 1 Laboratory of Proteomics and Microbiology, Research Institute for Biosciences, University of Mons 54521, Mons, Belgium; Freie Universitat Berlin, Berlin, Germany

**Keywords:** purple bacteria, metabolism, trophic link, resource recovery, microbial ecology

## Abstract

Purple non-sulfur bacteria (PNSB) are well known to have an exceptional metabolic versatility. However, while the growth of PNSB on sugar-rich streams has been extensively explored, their ability to metabolize sugars is poorly understood. Here, we explore the metabolic mechanisms of sucrose, glucose, and fructose utilization in two phototrophic PNSB, *Rhodospirillum rubrum* and *Rhodobacter capsulatus*. Our findings demonstrate distinct carbohydrate assimilation capacities, as well as the use of different metabolic strategies for each species. Moreover, a trophic link was identified between the two species during co-cultivation, resulting from the production of fermentation by-products by *Rh. capsulatus*, which are then reassimilated by *Rs. rubrum*. Finally, we demonstrate that the synergy observed between *Rs. rubrum* and *Rh. capsulatus* can be successfully scaled up in a photobioreactor system. Our study highlights how fundamental knowledge of metabolism and the establishment of a trophic link between two PNSB species might be useful for the development of biobased economy and resource recovery strategies.

**IMPORTANCE:**

The diverse metabolic capacities found in microbial communities expand the possibilities of microbial biotechnological exploitation. In this study, we demonstrate that *Rhodospirillum rubrum* and *Rhodobacter capsulatus*, two purple non-sulfur bacteria, adopt different metabolic strategies for sugar assimilation. These differences allow them to benefit from each other, resulting in enhanced carbon yield and productivity compared to pure cultures. We also showed that the trophic link between both species can be scaled up in a photobioreactor system. Understanding these interactions expands the potential for designing microbial consortia optimized for the valorization of carbohydrate-rich waste streams using purple non-sulfur bacteria.

## INTRODUCTION

For economic and ecological reasons, the circular economy has gained increasing attention in the last decades. One of the most promising technologies is the upcycling of liquid by-products of industrial processes rich in organic matter (cleaning water, whey, molasses, etc.) in high added-value components (carotenoids, microbial proteins, coenzyme Q10, etc.). Due to their well-known metabolic versatility, purple non-sulfur bacteria (PNSB) are good candidates for the development of such circular bioprocesses (1, 2). They are able to grow on a wide variety of carbon substrates, including alcohols (3), carbohydrates (4–6), or volatile fatty acids (7, 8). Purple non-sulfur bacteria can grow under a wide range of environmental conditions, adopting an aerobic chemotrophic metabolism in the presence of oxygen and a phototrophic metabolism in the presence of light and low oxygen tensions. Depending on the available carbon and electron sources, PNSB can assimilate CO_₂_ as the sole carbon source (autotrophy) or organic molecules (heterotrophy) (9). Therefore, PNSB are extensively studied for bio-industrial purposes (10), such as the treatment of wastewater (11), or the production of bioplastics (12), biohydrogen (13), or fertilizers (14). Purple bacteria are also shown to produce a biomass rich in both high-quality proteins (due to a good essential amino acid profile [15]) and health-promoting molecules such as carotenoids and coenzyme Q10 (15–18). This makes them good candidates for the production of microbial proteins for food and feed applications and the development of innovative protein sources (1).

Among the by-products of the food industry, sugar-containing streams are interesting as a source of carbon, as these molecules are generally easily assimilated by microorganisms. Molasses is one of the main co-products of the sugar industry obtained after sugar refining. Sugar beet molasses is mainly composed of sucrose (~61% [wt/wt] on a dry matter basis) but also contains low levels of other sugars, such as fructose, glucose, raffinose, galactose, and arabinose (<1% [wt/wt] on a dry matter basis), with the remaining fraction consisting of ash, organic acids, and crude proteins (19). The growth of PNSB on molasses or pure sugars has already been frequently reported, especially in the field of biohydrogen production, where the use of sugars can be employed in both dark fermentation and photofermentation processes (20–22). During dark fermentation, sugars are anaerobically fermented into organic acids; for example, *Rhodospirillum rubrum* (*Rs. rubrum*) has been reported to produce succinate, acetate, propionate (23), and formate (24), while *Rhodobacter capsulatus* (*Rh. capsulatus*) produces lactate, acetate, and succinate (24). Early studies investigated sugar assimilation pathways in *Rh. capsulatus* and *Rhodobacter sphaeroides* (5, 25), and to a lesser extent in *Rs. rubrum* (4), yet sugar assimilation under phototrophic conditions remains poorly understood, particularly in *Rs. rubrum*.

In this study, we analyzed how two model strains of PNSB (i.e*., Rs. rubrum* and *Rh. capsulatus*) thrive when fed sucrose as a main carbon source under phototrophic conditions. Assimilation of sucrose and its derivatives, fructose and glucose differed in the two strains, notably regarding sucrose hydrolysis capacity, as well as the proportion of sugars directed to fermentation. We highlighted a synergistic effect when the two strains were cultivated together on sucrose. Proteomic analyses revealed a trophic link between them, indicating that *Rs. rubrum* can grow on sucrose as the sole carbon source only when co-cultivated with *Rh. capsulatus*. Finally, our findings were used to implement a sequential batch photobioreactor cultivation strategy, paving the way to the development of PNSB-based molasses valorization strategy.

## MATERIALS AND METHODS

### Bacterial strains, medium composition, and culture conditions

*Rhodospirillum rubrum* S1H (ATCC 25903), the *ccr* knockout strain of *Rs. rubrum* S1H (Δ*ccr*::Km^r^ strain) (26), and *Rh. capsulatus* (ATCC 11166) were cultivated separately or in co-culture under anaerobic photoheterotrophic conditions. The carbon source of the culture medium was sucrose, glucose, or fructose or a mixture thereof, always provided at 120 mM total carbon equivalent. Nitrogen was provided as ammonium chloride (35 mM) and biotin (0.06 µM) as a vitamin. When required, the medium was supplemented with filtered NaHCO_3_ at 3 or 50 mM. *Rh. capsulatus* cultures were grown both with and without thiamine supplementation (0.89 µM), as thiamine is a required cofactor for pyruvate dehydrogenase. As it may influence the level of sucrose hydrolysis, the sterilization procedures as well as the complete culture medium composition are available in the supplemental material (see Supplemental Note, recipe and sterilization procedure). Stock cultures were grown in a supplemented malate-ammonium medium enriched with yeast extract and peptone (SMN medium) (see Supplemental Note, recipe and sterilization procedure).

Cultures were started in photoheterotrophy in 50 mL serum bottles filled with 40 mL working volume at a starting optical density at 680 nm (OD_680_) of 0.4 for pure cultures. Co-cultures were initiated from separate stock cultures of *Rs. rubrum* and *Rh. capsulatus*. The starting OD_680_ was set at 0.2 for each strain of bacteria. Depending on the experiment, the medium was previously autoclaved (resulting in partial hydrolysis of sucrose into fructose and glucose, with an average hydrolysis rate of 43.75% ± 7.43% based on residual sucrose quantification, *N* = 4) or filtered through a 0.22 µm filter for sterilization. After inoculation, oxygen was purged from the gas phase using a sterile nitrogen stream for 30 s. The bottles were then hermetically sealed to allow anaerobic growth of bacteria. Cultures were placed at 30°C, submitted to orbital shaking (170 rpm), and illuminated at 177 µmol photons m^−2^ s^−1^ with halogen lamps (10 W, 100 lm; 2,650 K; Sencys). The illumination intensity was corrected for detector bias (Apogee Quantum Flux MQ-200) using an in-house developed app (https://github.com/damien-dumont/LightConverter). The emission spectrum of halogen lamps can be found in the supplemental material (Table S1). Bacterial growth was monitored once a day by measuring OD_680_. A calibration curve of dry cell weight (DCW; mg/mL) in function of OD_680_ was used to determine biomass concentration for *Rs. rubrum* and *Rh. capsulatus* (Table S2). Maintenance of the axenic conditions was systematically confirmed after the completion of each experiment by plating the culture on SMN agar plates.

Carbon yield was calculated as follows: biomass concentration was determined from OD_680_ measurements, assuming that an OD_680_ of 1 corresponds to 0.6 g/L of dry biomass (Table S2). The molecular formula of the biomass was assumed to be C_5_H_7_O_2_N, corresponding to five carbon atoms per mole of biomass. The biomass carbon content (in mmol) was calculated accordingly, and the carbon yield was determined as the ratio of carbon converted into biomass to the total amount of assimilated carbon (mmol C in biomass/mmol of assimilated C substrate). Productivity was calculated by converting the maximum OD_680_ reached by the culture into g/L using the calibration curve (Table S2), then dividing by the number of days in culture corresponding to that maximum OD_680_ to obtain a productivity in g/L·day. The efficiency of sucrose utilization for biomass production was calculated as the ratio of the biomass produced (g/L, dry weight) to the amount of sucrose supplied in the medium (g/L).

### Culture conditions in photobioreactors

Prior to cultivation in the photobioreactors, *Rs. rubrum* and *Rh. capsulatus* were cultivated (i.e*.*, pre-cultures) as previously described in sealed serum bottles using an SMN medium. For the sequential batch photobioreactor (PBR) experiment, *Rs. rubrum* and *Rh. capsulatus* (in pure or mixed cultures) were cultivated in a 2 L glass vessel (Biostat, Sartorius AG, Germany). The cultures were illuminated continuously with a 20 W light source (201 lumens, 2,700 K, Sencys), providing an intensity of ±400 µmol photons m⁻² s⁻¹ (Li-205A, Li-Cor BioSciences). Agitation was set to 100 rpm. Light intensity was adjusted for detector bias using a custom app (https://github.com/damien-dumont/LightConverter), with halogen lamp emission spectra available in Table S1.

The carbon source of the culture medium was sucrose at a concentration of 120 mM carbon equivalent. Nitrogen was supplied as ammonium chloride (35 mM), and biotin (0.06 µM) as a vitamin following the protocol outlined in Bayon-Vicente et al. (27). Starting OD_680_ was set at ~0.4. To conduct sequential batches, 90% of the culture was replaced by fresh medium once the stationary phase was reached, defined by two consecutive stable OD_680_ readings. In the case of the addition of a strain (i.e*.*, *Rh. capsulatus*) during sequential batch PBR, the pre-culture was added to the fresh medium under a laminar flow hood and transferred into the photobioreactor. As for serum bottle experiments, maintenance of the axenic conditions of the cultures was confirmed after each batch cycle by plating the culture on SMN agar plates.

### Monitoring of sugar consumption

Culture samples were centrifuged at 10,000 × *g* speed for 10 min to eliminate biomass. Sucrose, fructose, and glucose were analyzed using liquid chromatography-mass spectrometry (LC-MS) analyses. Sugars were separated using an HPLC system (Sciex, ExionLCTM Series UHPLC, Shimadzu model) on an XBridge BEH Amide column (130 Å, 2.1 × 150 mm, 5 μm) (Waters Corporation, 186006590). The separation was carried out in isocratic mode, with a flow rate of 0.6 mL/min at 80°C. The mobile phase was a mix of acetonitrile and 5 mM ammonium formate at pH 7 (85:15, vol/vol). The separation was completed in 7 min, and the injection volume was 1 µL. Samples were analyzed online with a ZenoTOF 7600 mass spectrometer (SCIEX). MS spectra were collected using the ZenoTOF 7600 system between 20 and 400 Da with the following parameters: ionization was performed in negative electrospray ionization (ESI) mode, the ion source temperature was set to 250°C, the capillary voltage was set to −4,500 V, the declustering potential was set to −80 V, and the collision energy was set to −5 V.

SCIEX OS software (version 2.1.6.59781) was used for sugar quantification by using a quantitation and targeted identification method. Glucose, fructose, and sucrose were quantified by integrating their specific peaks (retention times of 1.867, 1.483, and 3.447 min, respectively) in comparison with a reference curve constructed with standards.

### Monitoring of organic acid and ethanol production

Culture samples were centrifuged at 10,000 × *g* speed for 10 min to eliminate biomass. Fifty microliters of the sample was transferred to a reaction tube. Subsequently, 25 µL of 200 mM 3-nitrophenylhydrazine hydrochloride was added, followed by 25 µL of 50 mM 1-ethyl-3-(3-dimethylaminopropyl)carbodiimide (EDC) and 25 µL of a 7% (vol/vol) pyridine solution. The mixture was gently vortexed and incubated at 30°C for 5 h with shaking at 500 rpm. The derivatization reaction was stopped by adding 125 µL of 5% (vol/vol) formic acid. Derivatized organic acids were analyzed using LC-MS. Separation was performed using an HPLC system (Sciex, ExionLCTM Series UHPLC, Shimadzu model) equipped with a F5 column (100 Å, 2.1 × 150 mm, 5 μm) (Phenomenex, Kinetex, 00F-4724-AN). The separation was carried out using a gradient elution with the following conditions: the flow rate was set to 0.2 mL/min, and the column temperature was maintained at 40°C. The chromatography consisted of a gradient between phase A (H_₂_O + 0.1% ammonium formate) and phase B (acetonitrile + 0.1% ammonium formate), with phase B held at 35% for the first 5 min, then increased to 90% by 6.9 min, and subsequently decreased to 10% by 7.5 min. The injection volume was 5 µL. Samples were analyzed online with a ZenoTOF 7600 mass spectrometer (SCIEX). MS spectra were acquired in the *m/z* range of 100 and 1,000 Da under the following parameters: ionization was performed in ESI mode, with an ion source temperature of 300°C. The capillary voltage was set to 5,500 V, the declustering potential to 80 V, and the collision energy to 10 V. SCIEX OS software (version 2.1.6.59781) was used for organic acid quantification by using a quantitation and targeted identification method. Hexanoate, propionate, acetate, lactate, succinate, butyrate, citrate, and hippurate were quantified by integrating their specific peaks (retention time of 4.74, 3.245, 2.99, 2.86, 2.88, 3.57, 2.937, and 5.537 min, respectively) in comparison with a reference curve constructed with standards.

Formic acid and ethanol were additionally quantified using a colorimetric method with a detection kit (MAK491, Sigma-Aldrich, for formic acid quantification and MAK481-1KT, Sigma-Aldrich, for ethanol quantification). Culture samples were centrifuged at 10,000 × *g* speed for 10 min, and formic acid quantification was performed on the supernatants following the manufacturer’s instructions.

### Strain quantification

Results from proteomic analyses were used as a proxy for strain quantification, as it was shown to be more robust than regular methods such as plating and 16S rRNA sequencing (Data S1). Bacteria were collected through centrifugation (10,000 × *g*, 10 min). Proteins were extracted from the pellet using guanidine hydrochloride 6 M assisted by ultrasonication (pulse 1, amplitude 20%, 3 × 10 s) (IKA U50 control instrument). Samples were centrifuged at 10,000 × *g,* and protein concentration was determined using the Bradford method with bovine gamma globulin as a standard. Fifty micrograms of proteins were then reduced (with 25 mM DTE), alkylated (with 50 mM iodoacetamide), and overnight precipitated (with cold acetone 80% [vol/vol]). Proteins were recovered through centrifugation at 13,000 × *g* at 4°C for 20 min, and the supernatant was discarded. Proteins were resuspended in 25 mM bicarbonate solution and trypsinized overnight at 37°C at a 1/50 enzyme/substrate ratio. The digestion was stopped by adding formic acid (0.1% [vol/vol] final concentration).

MS data were acquired using data-dependent acquisition mode (DDA) on a UHPLC HRMS platform (Eksigent 2D ultra-AB SCIEX TripleTOF 6600). A 15 cm C18 column (YMC-Triat 0.3 × 150 mm column) was used to separate 2 µg of trypsin-generated peptides. Peptides were separated with a linear acetonitrile (ACN) gradient (5%–35% [vol/vol] in 20 min) in water containing 0.1% (vol/vol) formic acid at a flow rate of 5 µL/min. MS survey scans (*m/z* 400–1,250, 100 ms accumulation time) were followed by 50 MS/MS acquisitions of the most abundant doubly or triply charged precursors (exclusion after 2 occurrences for 12 s). Collision-induced dissociation was carried out using rolling collision energy, and fragment ions were accumulated for 50 ms in high-sensitivity mode. MS/MS data were processed with ProteinPilot software (version 5.0.1.0,4895, AB SCIEX) and analyzed against databases containing the proteomes of *Rs. rubrum* and *Rh. capsulatus*. Carbamidomethylation of cysteine and all biological modifications and amino acid substitutions were used as fixed and variable parameters for database search, respectively. The results were then used to quantify the number of proteins identified for each strain separately and calculate their proportions in relation to the number of total identified proteins. This proportion was used as a proxy of the strain proportion in the culture.

### Differential proteomic analysis

For quantitative proteomic analyses, samples were prepared as described above. Five micrograms of tryptic peptides were separated on a C18 LC as described above, using a 75 min 3%–35% ACN gradient. TripleTOF 6600 was used in data-independent acquisition mode (SWATH). For SWATH analyses, 100 incremental steps were defined as windows of variable *m/z* values over a 400–1,250 *m/z* mass range. The MS/MS accumulation time for each SWATH window was 50 ms, leading to a duty cycle of 5 s per cycle. Peptides were identified using in-house-produced spectral library created in DDA mode (as described above, but with 75 min ACN gradient) with biomass obtained in all relevant conditions (carbon sources and co-cultures). For peptide quantification, extracted ion chromatograms were extracted with the Skyline software (23.1) for their six most abundant fragments, and their area under the curve was integrated and summed. Skyline was used for the quantification of proteins identified at an FDR below 0.95% (as determined by ProteinPilot). For co-culture conditions, *Rs. rubrum* and *Rh. capsulatus* were processed separately on Skyline using their specific proteome fasta files. Processing data separately for the different strains allowed normalization of the data at the strain level and consideration of differences in strain relative abundances in co-cultures and in pure cultures. Only proteins quantified with two or more peptides, with a fold change higher than 1.5 or lower than 0.66 and having a *P*-value lower than 0.05, were further considered (raw data are provided in supplemental tables and analysis files).

### Statistics

Experiments were carried out by using three or five replicates for each condition. The graphs and statistical analyses were carried out using GraphPad software (GraphPad Prism 8, version 8.0.1). Skyline packages were used for statistical analyses of the protein quantitative data. Skyline’s Group Comparison uses *t*-test to calculate *P-*values for differences in protein abundances between conditions.

## RESULTS AND DISCUSSION

### Sucrose assimilation by *Rs. rubrum* and *Rh. capsulatus*

Several studies using molasses as a nutrient source, or a synthetic medium containing sucrose, have already been carried out on PNSB (16, 20, 28). It is, therefore, commonly accepted that PNSB can assimilate sucrose. However, in all these previous studies, when the medium sterilization procedure was mentioned, it was carried out through autoclaving, which leads to the partial hydrolysis of sucrose into glucose and fructose. We thus wondered whether purple bacteria are effectively capable of assimilating sucrose or if reported growth relied on the presence of fructose and glucose in the culture medium. Therefore, we cultivated two model species of PNSB, *Rs. rubrum* and *Rh. capsulatus*, in a sucrose-containing medium sterilized through filtration, avoiding sucrose hydrolysis.

**Fig 1:**
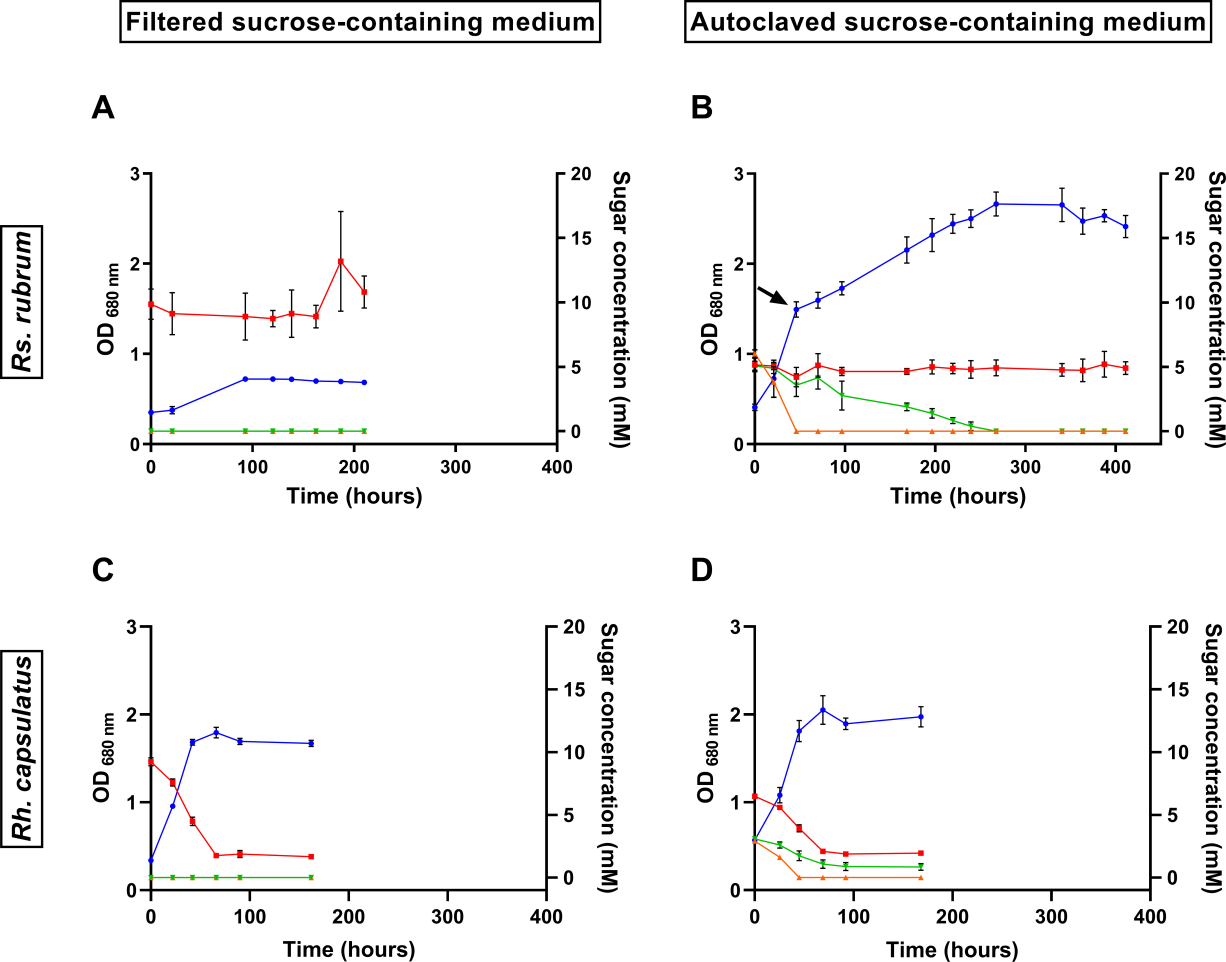
Monitoring of growth (blue line) of *Rs. rubrum* (**A and B**) and *Rh. capsulatus* (**C and D**) cultivated in a medium containing sucrose previously filtered (**A and C**) or autoclaved (leading to partial hydrolysis of sucrose) (**B and D**) and illuminated at 177 µmol photons m^−2^ s^−1^. Red, green, and orange lines represent the evolution of sucrose, glucose, and fructose concentration, respectively. The black arrow represents a sampling time for proteomic analysis. *n* = 5. Results are represented as the mean ± SD.

After 200 h of incubation, *Rs. rubrum* only reached an OD_680_ of 0.7 ± 0.02 (0.42 ± 0.01 mg DCW/mL) in the sucrose-containing medium, the concentration of which remained stable along the monitoring time as determined through LC-MS measurement (Fig. 1A). These results suggest that *Rs. rubrum* is unable to assimilate sucrose, and the observed limited increase in OD is probably due to the mobilization of carbon storage accumulated in the pre-culture phase, as the transfer of some nutrients with the inoculum is unlikely, since the inoculum was washed before inoculation to limit nutrient transfer. An additional possibility is that the observed limited growth could partly result from autotrophic assimilation of dissolved CO_₂_ using trace electron donors present in the medium. In contrast, *Rh. capsulatus* grew up to an OD_680_ of 1.7 ± 0.04 (1.0 ± 0.02 mg DCW/mL) and almost completely consumed sucrose, demonstrating that this species is capable of assimilating sucrose (Fig. 1C). Interestingly, the carbon yield (grams of produced biomass per gram of consumed carbon source) is much lower than what we usually observe with volatile fatty acids fed at the same initially equivalent carbon levels (120 mM) and for which an OD_680_ of up to 4 is usually observed (27, 29) (for easier comparison, biomass production, carbon yield, and productivity data from the experiments discussed in this study have been summarized in Table S3). Notably, neither fructose nor glucose release was observed in the culture medium, suggesting sucrose hydrolysis occurs intracellularly or that glucose and fructose are rapidly reassimilated to a non-detectable level.

To investigate the growth dynamics of *Rh. capsulatus* and *Rs. rubrum* on a sucrose-containing medium, we cultivated both strains with partially hydrolyzed sucrose obtained after autoclaving the culture medium. Despite not being able to assimilate sucrose, *Rs. rubrum* reached an OD_680_ of 2.4 ± 0.1 (1.4 ± 0.07 mg DCW/mL) (Fig. 1B). We observed that both fructose and glucose (generated through thermal hydrolysis of sucrose) were fully assimilated, although several studies claimed that *Rs. rubrum* was not capable of assimilating glucose (4, 30). Interestingly, fructose was assimilated more rapidly than glucose in *Rs. rubrum*, leading to a higher productivity (0.45 ± 0.03 g/L·day) compared to that observed during glucose assimilation (0.07 ± 0.01 g/L·day) (Fig. 1B). This difference in assimilation rates was consistent with the biphasic growth pattern of *Rs. rubrum*, which showed a rapid initial growth associated with fructose utilization, followed by a slower growth phase corresponding to glucose assimilation when all the fructose was consumed. Differences in the assimilation rates of various carbon sources have already been observed in several bacterial species (reviewed by Görke and Stülke [31]) and also in PNSB, as reported by our laboratory, in the case of volatile fatty acid assimilation (7). A similar phenomenon has been observed with the uptake of sugars in PNSB, where a preference for glucose over xylose was noted in a species of the genus *Rhodobacter* (32). However, a preferential assimilation of fructose over glucose in *Rs. rubrum* has not yet been demonstrated, as this strain has been described as not capable of using glucose as the sole carbon source (4, 30). Notably, in our conditions, glucose was only assimilated if provided in combination with fructose, whereas little to no glucose assimilation was observed (productivity of 0.02 g/L·day) when it was the sole carbon source, which is in line with previous reports (Fig. S1).

As a lack of membrane transport could be the reason for the usually reported inability of *Rs. rubrum* to assimilate glucose, we wondered if potential glucose transporters were upregulated during the glucose assimilation phase. To explore this, we compared the proteome of *Rs. rubrum* during fructose uptake with its proteome during glucose uptake, which occurred when all the fructose was consumed. *Rs. rubrum* was cultivated in a medium containing fructose and glucose (50/50) as carbon sources (Fig. S2). Results showed that three proteins known to be involved in sugar transport showed a significantly higher relative abundance in the glucose assimilation phase than in the fructose assimilation phase. Fold change values < 1 are reported here as negative values (–1/FC) to indicate decreased abundance relative to the reference. The extracellular solute-binding protein, family 1 (Rru_A0092), was detected with a FC of −2.94 (*P* = 7.08 × 10⁻^6^). The ribose ABC transporter, periplasmic binding protein (Rru_A1365), was detected with a FC of −2.22 (*P* = 1.7 × 10⁻^3^), and the periplasmic binding protein/LacI transcriptional regulator (Rru_A1336) was detected with a FC of −3.70 (*P* = 2.9 × 10⁻^3^). The first two proteins mentioned were automatically annotated as potential glucose transporters, but to date, no evidence of their role in glucose transport has been demonstrated. On the other hand, the LacI-family transcriptional regulators are known to regulate sugar transport, particularly that of glucose (33). These results might suggest that some of these proteins are involved in glucose transport across the cytoplasmic membrane. Furthermore, we also showed that the PTS fructose IIC component (Rru_A1970) was detected in higher abundance during the fructose assimilation phase (FC of 1.86, *P* = 2.9 × 10⁻^4^) (the complete data set is available in Table S4). This result corroborates previous findings showing that fructose is assimilated by a phosphoenolpyruvate-sugar phosphotransferase system (PTS) (30) but also suggests that glucose is either not transported or less efficiently transported by the same transporter as fructose.

When sucrose was partially hydrolyzed, *Rh. capsulatus* was able to assimilate fructose, glucose, and sucrose, reaching an OD_680_ of 2.05 ± 0.16 (1.23 ± 0.10 mg DCW/mL) (Fig. 1D). However, glucose and sucrose were not completely assimilated. Incomplete uptake of sucrose has also been observed for *Rh. capsulatus* in a medium containing non-hydrolyzed sucrose (Fig. 1C). By comparing the carbon yield in all conditions, we observed that it was higher for *Rs. rubrum* on partially hydrolyzed sucrose (1.0 mmol C in biomass/mmol of assimilated C substrate) than for *Rh. capsulatus* either on partially hydrolyzed sucrose (0.50 mmol C in biomass/mmol of assimilated C substrate) or on non-hydrolyzed sucrose (0.43 mmol C in biomass/mmol of assimilated C substrate).

Taken together, the growth profiles, substrate utilization, and carbon yields strongly suggest that both strains mainly grow photoheterotrophically in the presence of carbohydrates as the carbon source and under continuous illumination, since no terminal electron acceptor was supplied and respiration can, therefore, be theoretically excluded. Although a minor portion of ATP is generated through substrate-level phosphorylation (limited to roughly two ATP molecules per hexose via the Embden-Meyerhof-Parnas [EMP]/ED pathways and fermentation to acetate), the majority of ATP is expected to originate from photophosphorylation. However, the metabolic routes used to assimilate carbohydrates appear to differ between *Rs. rubrum* and *Rh. capsulatus*. In particular, differences in substrate partitioning between the EMP and ED pathways, in the levels of organic acids produced, and in the reassimilation of CO_₂_ released during oxidative steps may account for the higher carbon yield observed in *Rs. rubrum* compared to *Rh. capsulatus*.

### Carbohydrate (photo-)assimilation by *Rs. rubrum* and *Rh. capsulatus*

**Fig 2:**
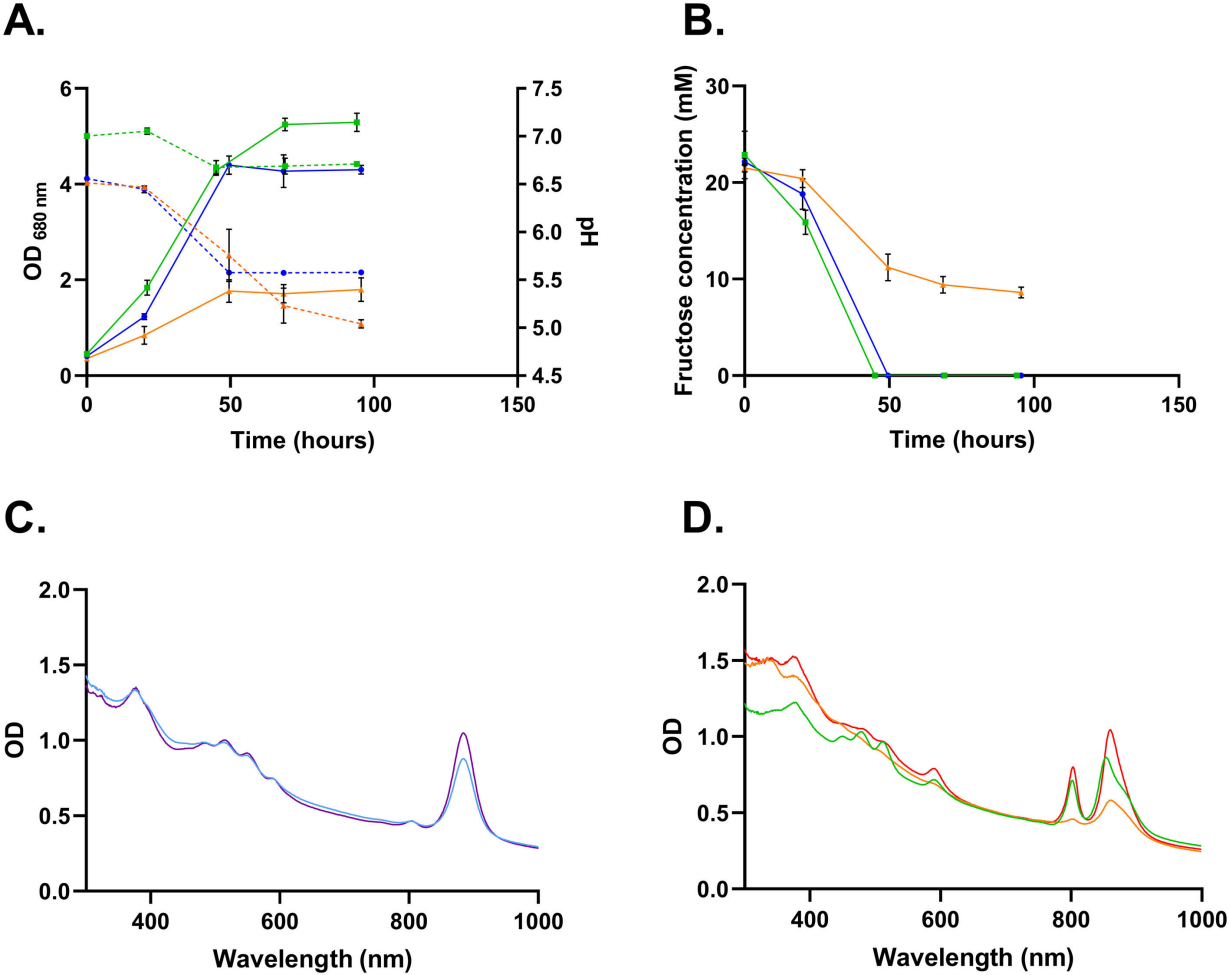
Growth, pH evolution, and absorbance spectra of *Rs. rubrum* and *Rh. capsulatus* cultivated on fructose. (**A**) Monitoring of growth (solid lines) and pH evolution (dotted lines) of *Rs. rubrum* (blue line) and *Rh. capsulatus* grown with (green line) or without (orange line) thiamine supplementation in a fructose-containing medium and illuminated at 177 µmol photons m^−2^ s^−1^. (**B**) Fructose concentration during growth for *Rs. rubrum* (blue line) and *Rh. capsulatus* with and without thiamine (green and orange lines, respectively). (**C**) Absorbance spectra of *Rs. rubrum* grown on fructose (blue line) or succinate (purple line) after 50 h. (**D**) Absorbance spectra of *Rh. capsulatus* grown on fructose with or without thiamine (green and orange lines, respectively) or succinate (red line) after 50 h. *n* = 3 for panels A and B. Results are represented as the mean ± SD. *n* = 1 for panels C and D.

To compare sugar assimilatory metabolism in *Rs. rubrum* and *Rh. capsulatus*, both species were first cultivated separately in a medium containing 20 mM fructose as the sole carbon source, which both species can readily assimilate, in contrast to sucrose and glucose. Importantly, *Rh. capsulatus* was grown both with and without thiamine supplementation (0.89 µM). Indeed, thiamine is a required cofactor of pyruvate dehydrogenase, the enzyme that catalyzes the oxidative decarboxylation of pyruvate to acetyl-CoA. It has been described that *Rh. capsulatus* requires thiamine supplementation, indicating a lack of the enzymatic machinery necessary for thiamine production, while *Rs. rubrum* possesses it (34). This allows a proper comparison between the prototrophic growth of *Rs. rubrum* and *Rh. capsulatus*. Under these conditions, *Rs. rubrum* reached an OD_680_ of 4.5 ± 0.17 (2.7 ± 0.1 mg DCW/mL), while *Rh. capsulatus* with thiamine reached 5.3 ± 0.19 (3.2 ± 0.11 mg DCW/mL), compared to only 1.8 ± 0.25 (1.0 ± 0.15 mg DCW/mL) for *Rh. capsulatus* without thiamine (Fig. 2A, solid lines).

pH monitoring revealed that in prototrophic conditions (*Rs. rubrum* and *Rh. capsulatus* with thiamine), the pH drop was limited, from 6.6 to 5.5 in *Rs. rubrum* and from 7.0 to 6.7 in *Rh. capsulatus*, whereas in *Rh. capsulatus* without thiamine, pH decreased more sharply from 6.5 to 5.0 (Fig. 2A, dotted lines). This demonstrates that thiamine availability strongly affects acidification of the medium in *Rh. capsulatus* cultures. The limited pH drop in *Rh. capsulatus* with thiamine reflects efficient sugar assimilation and a lower accumulation of acidic intermediates, while the larger pH decrease in the absence of thiamine is likely due to a metabolic bottleneck caused by insufficient activity of the pyruvate dehydrogenase. This excess pyruvate can increase flux through key fermentative enzymes, including lactate dehydrogenase, pyruvate:ferredoxin oxidoreductase, and pyruvate formate-lyase, leading to the production of a mixture of organic acids. Fructose consumption mirrored growth patterns (Fig. 2B). *Rs. rubrum* and *Rh. capsulatus* with thiamine fully consumed fructose within ~50 h, whereas *Rh. capsulatus* without thiamine only assimilated ~50% of the substrate. Consistent with these observations, the carbon yield was 0.95 for *Rs. rubrum* on fructose and 1.0 for *Rh. capsulatus* when supplied with thiamine, while a reduced value of 0.67 was measured in cultures without thiamine.

The absorption spectra were obtained after 50 h of culture, and all samples were adjusted to an OD_680_ of 0.5 prior to measurement to allow direct comparison of pigment content. Clear differences in culture coloration were also visible to the naked eye in *Rh. capsulatus* cultures without thiamine supplementation, supporting that the observed variations in pigment spectra reflect actual differences in cellular pigment levels (Fig. S3). Absorbance spectra showed clear differences in photosynthetic pigments depending on carbon source (Fig. 2C and D). Both species displayed reduced pigment content in fructose without thiamine supplementation compared to a control condition with succinate as a carbon source, indicating decreased phototrophic metabolism in the fructose-containing medium. This phenomenon was observed in both species, although it was more pronounced in *Rh. capsulatus* grown without thiamine. In contrast, thiamine-supplemented *Rh. capsulatus* showed a spectrum similar to that with succinate, indicating that pigment levels were maintained.

To investigate the origin of the pH drop, several organic acids (hexanoate, propionate, acetate, lactate, succinate, butyrate, citrate, and formate) were quantified in both *Rs. rubrum* and *Rh. capsulatus* culture supernatants after 70 h of cultivation (Table S5). Notably, in the absence of thiamine, *Rh. capsulatus* accumulated significant amounts of formate (~4–5 mM) and lactate (~2–3 mM), along with low concentrations of acetate and butyrate (<1 mM). In thiamine-supplemented cultures, some of the quantified organic acids showed significant differences compared to cultures without thiamine; however, their concentrations remained low with approximately 1.5 mM formate, 2 mM lactate, and about 1 mM each of acetate and butyrate detected (Fig. S4). In *Rs. rubrum*, these compounds were mostly undetectable, although a pH decrease of 0.5–0.8 units was observed, suggesting the secretion of other, today unknown, organic compounds. Because our analysis focused on selected fermentation products, the observed acidification may also result from the production of other organic acids that were not quantified in this study. Taken together, these observations indicate that both *Rs. rubrum* and *Rh. capsulatus* appear to rely on mixed-acid fermentation under the tested anaerobic and illuminated conditions, although the specific organic acids produced differ between the two species. In *Rh. capsulatus*, the absence of thiamine strongly limits pyruvate dehydrogenase activity, resulting in a pronounced pH drop and incomplete sugar assimilation. The presence of thiamine allowed complete sugar assimilation with a much less pronounced pH decrease. As similar levels of acids were detected in cultures with or without thiamine, this suggests that, as in *Rs. rubrum*, other organic acids may be produced when thiamine is absent.

Under phototrophic conditions, the activation of mixed-acid fermentation pathways may also be a response to redox stress caused by the accumulation of reducing equivalents (e.g., NADH) produced through the EMP pathway. To test this hypothesis, we supplemented *Rh. capsulatus* and *Rs. rubrum* cultures with 50 mM bicarbonate ions, which are known to allow electron sinking through CO_2_ fixation in PNSB (35). The addition of bicarbonate ions increased the initial pH of the medium due to their buffering capacity. Although the magnitude of the pH drop (ΔpH) was significantly smaller at 50 mM compared to 0 and 3 mM bicarbonate ions (ANOVA, *P* < 0.05), the final optical density reached was not significantly different among conditions (ANOVA, *P* > 0.05) (Fig. S5). Therefore, bicarbonate ion supplementation did not alter the growth behavior of *Rh. capsulatus* and *Rs. rubrum*, indicating that the activation of mixed-acid fermentation pathways is unlikely to result from redox stress. Finally, fermentation might also be triggered by light limitation in the cultures, and further experiments are required to explore this possibility. However, as indicated by the pH drop (Fig. 2A), fermentation is observed from the very beginning of growth, suggesting it is not triggered by light limitation.

**Fig 3:**
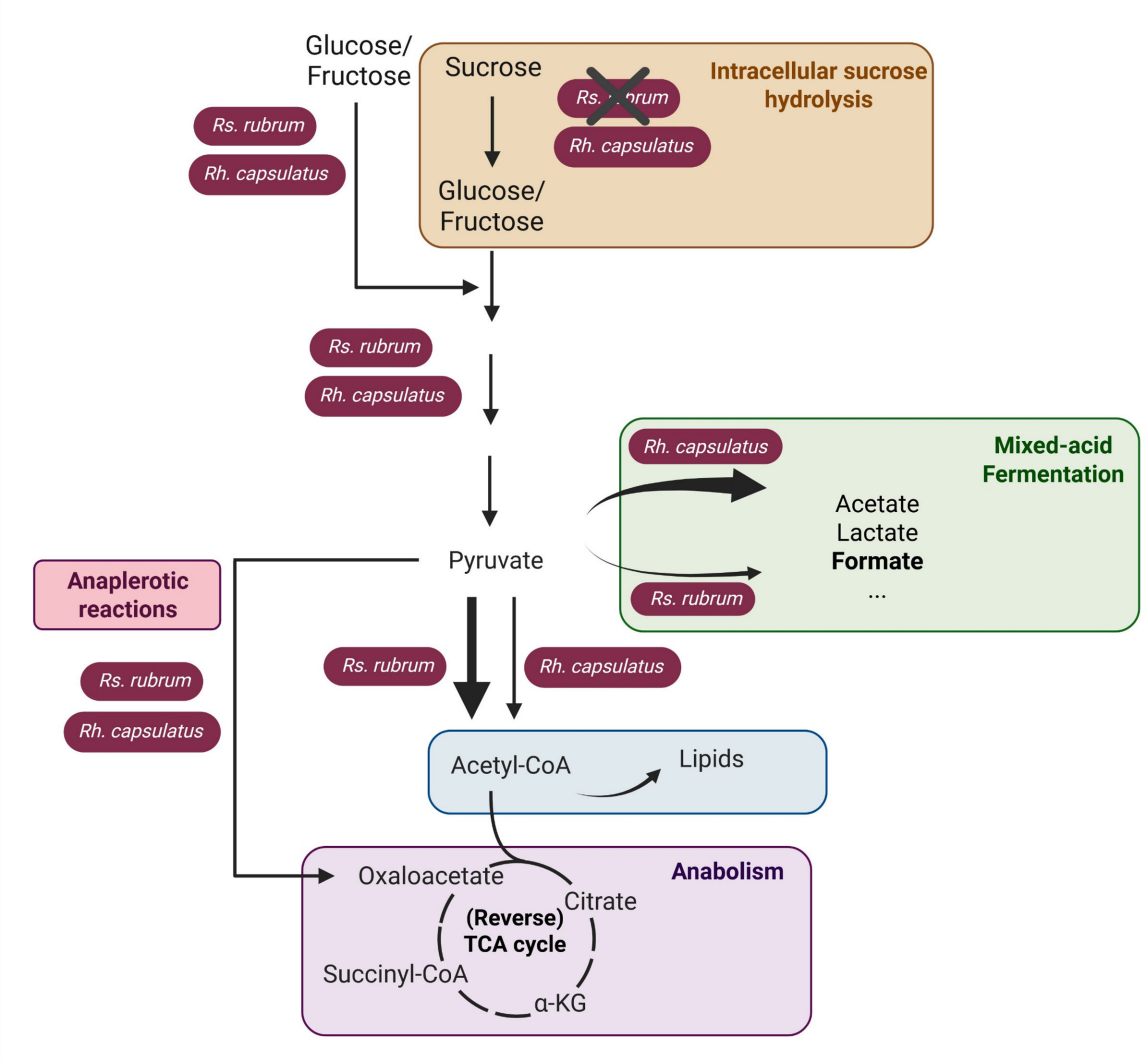
Schematic representation of potential glucose/fructose/sucrose assimilation pathways used by *Rs. rubrum* and *Rh. capsulatus* in photoheterotrophy under anaerobic conditions and without thiamine supplementation. *Rh. capsulatus* hydrolyzes sucrose into glucose and fructose, whereas *Rs. rubrum* lacks this ability. Glycolysis converts glucose and fructose to pyruvate, which is then metabolized differently in the two species. In *Rs. rubrum*, there is no limitation in pyruvate conversion to acetyl-CoA. In *Rh. capsulatus*, a large fraction of pyruvate is converted into fermentation products such as lactate, formate, and acetate due to limitation in the activity of pyruvate dehydrogenase in the absence of thiamine. The TCA cycle is used here solely to generate metabolic intermediates. Created in BioRender (M. Gilson, 2025).

These results emphasize that the minor genetic differences existing between *Rs. rubrum* and *Rh. capsulatus* can lead to major differences in sugar assimilation capacities, particularly when sufficient vitamins are not available, thus also influencing their potential biotechnological applications (Fig. 3). Based on the observed variation in sugar assimilation strategies in *Rs. rubrum* and *Rh. capsulatus,* we wondered whether a synergy might exist between them in co-culture.

### Synergistic effect in sucrose utilization in purple non-sulfur bacteria

The use of microbial mixed cultures can present an advantage over pure cultures thanks to the presence of a higher degree of metabolic diversity. Studies are thus often considering the use of synthetic consortium or natural consortium-based systems rather than pure culture systems (36–39). In this context, the use of a consortium of PNSB or PNSB-enriched natural communities has increased significantly in recent years. The applications of these studies are varied and include hydrogen production (40), wastewater treatment (37), polyhydroxyalkanoates (36), microbial protein production (41), etc.

**Fig 4:**
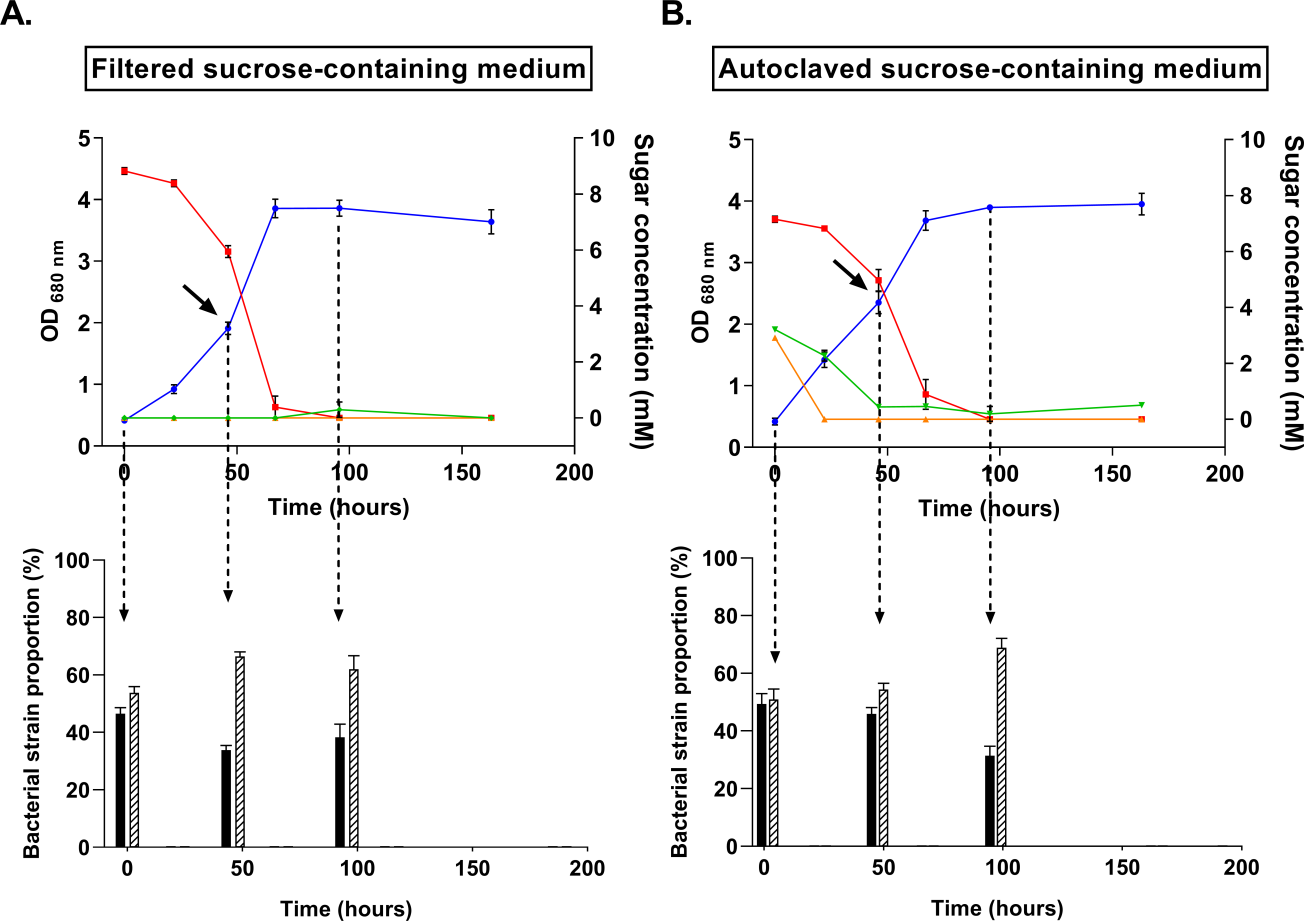
Monitoring of growth (blue line) of co-cultures *Rs. rubrum*/*Rh. capsulatus* grown on either previously filtered (**A**) or autoclaved (**B**) sucrose-containing medium and illuminated at 177 µmol photons m^−2^ s^−1^. Bar plots represent the proportions of *Rs. rubrum* (black bars) and *Rh. capsulatus* (hatched bars) at different time points. Red, green, and orange lines represent the evolution of sucrose, glucose, and fructose concentration, respectively. Black arrows represent sampling times. *n* = 5. Results are represented as the mean ± SD.

In the presence of partially hydrolyzed sucrose, our results suggested that *Rh. capsulatus* can use sucrose, glucose, and fructose, while *Rs. rubrum* can only use glucose and fructose but achieved a better growth yield. Moreover, we also showed that both species adopt distinct metabolic pathways when cultivated in the presence of fructose. We, therefore, investigated whether a *Rs. rubrum*/*Rh. capsulatus* co-culture could improve the assimilation efficiency and the growth yield of the different carbohydrates contained in the medium. The co-culture inoculated in a medium containing only filtered sucrose as a carbon source reached an OD_680_ value of 3.6 ± 0.2 (2.2 ± 0.12 mg DCW/mL) and a carbon yield of 0.82 mmol C in biomass/mmol assimilated C substrate. Such a high bacterial biomass production could not be reached when we used pure cultures of either *Rs. rubrum* or *Rh. capsulatus* in media without thiamine supplementation. Notably, the sucrose was fully assimilated by the co-culture (Fig. 4A), in contrast to what we observed with the individual strains (Fig. 1A and C). The sucrose conversion yield was largely improved in co-cultures compared to pure cultures. In pure cultures, the final biomass reached 0.42 mg DCW/mL for *Rs. rubrum* and 1 mg DCW/mL for *Rh. capsulatus*, whereas the biomass reached 2.2 mg DCW/mL in co-cultures grown in a filtered sucrose-containing medium. Moreover, the presence of *Rs. rubrum* stimulated the growth of *Rh. capsulatus*, with its biomass reaching 1.36 mg DCW/mL at the end of the growth in co-cultures, compared to 1 mg DCW/mL in pure cultures. A similar result could be observed for co-cultures grown in a partially hydrolyzed sucrose-containing medium. In this condition, the co-culture reached an OD_680_ value of 4.0 ± 0.18 (2.4 ± 0.11 mg DCW/mL), and all carbohydrates were also fully assimilated (Fig. 4B), showing a carbon yield of 0.84 mmol C in biomass/mmol C assimilated substrate. Importantly, in both conditions, pH monitoring revealed that the acidification of the medium was strongly limited in co-cultures (Fig. S6) compared to *Rh. capsulatus* cultures (Fig. 2A), indicating that *Rs. rubrum* helped maintain the pH.

To determine which strain thrives the best in the co-culture and is responsible for higher conversion yield, we investigated the proportions of *Rs. rubrum* and *Rh. capsulatus* using a proteomic-derived method (see method validation in Data S1). Surprisingly, while we have previously shown that this strain was unable to assimilate non-hydrolyzed sucrose, *Rs. rubrum* was still representing around 40% of the total bacterial population (Fig. 4A, bar graph), producing an estimated amount of biomass of 0.86 mg DCW/mL in the presence of sucrose only. This observation of a synergistic effect in the co-culture suggests a trophic link between the two PNSB strains.

### Trophic link between *Rs. rubrum* and *Rh. capsulatus* in co-culture

To explain how *Rs. rubrum* acquires the capacity to grow on sucrose when in co-culture with *Rh. capsulatus*, we hypothesized that the latter hydrolyzed sucrose, releasing fructose and glucose to the medium, thus enabling *Rs. rubrum* to grow by assimilating these two monosaccharides. However, we did not detect fructose in the culture medium. Moreover, based on the previously determined low assimilation rate of glucose in *Rs. rubrum,* the hydrolysis of sucrose should have led to the accumulation of glucose in the culture medium; however, only a very limited amount of glucose was detected (Fig. 4A, <1 mM). These results support the hypothesis proposing that sucrose hydrolysis occurs intracellularly in *Rh. capsulatus* during growth on this carbon source. Alternatively, and as suggested by our previous experiment showing that *Rh. capsulatus* switched to a fermentative metabolism during sucrose assimilation, organic acids detected in the medium could have been used as carbon sources by *Rs. rubrum*. Such a cross-feeding phenomenon between *Rs. rubrum* and *Rh. capsulatus* would make the co-culture system more advantageous than pure cultures for improving PNSB biomass production with carbohydrates as carbon sources.

To better understand how *Rs. rubrum* thrives on sucrose when cultivated with *Rh. capsulatus*, we carried out proteomic analyses to compare the proteome of *Rs. rubrum* in pure cultures with a previously autoclaved sucrose-containing medium (thus containing sucrose, fructose, and glucose) with its proteome in co-cultures with *Rh. capsulatus,* either in autoclaved or filtered sucrose-containing medium. Sampling times are indicated by black arrows in Fig. 1B and 4. Proteomic data normalization used the differential relative abundance of *Rs. rubrum* in pure and mixed cultures.

**Fig 5:**
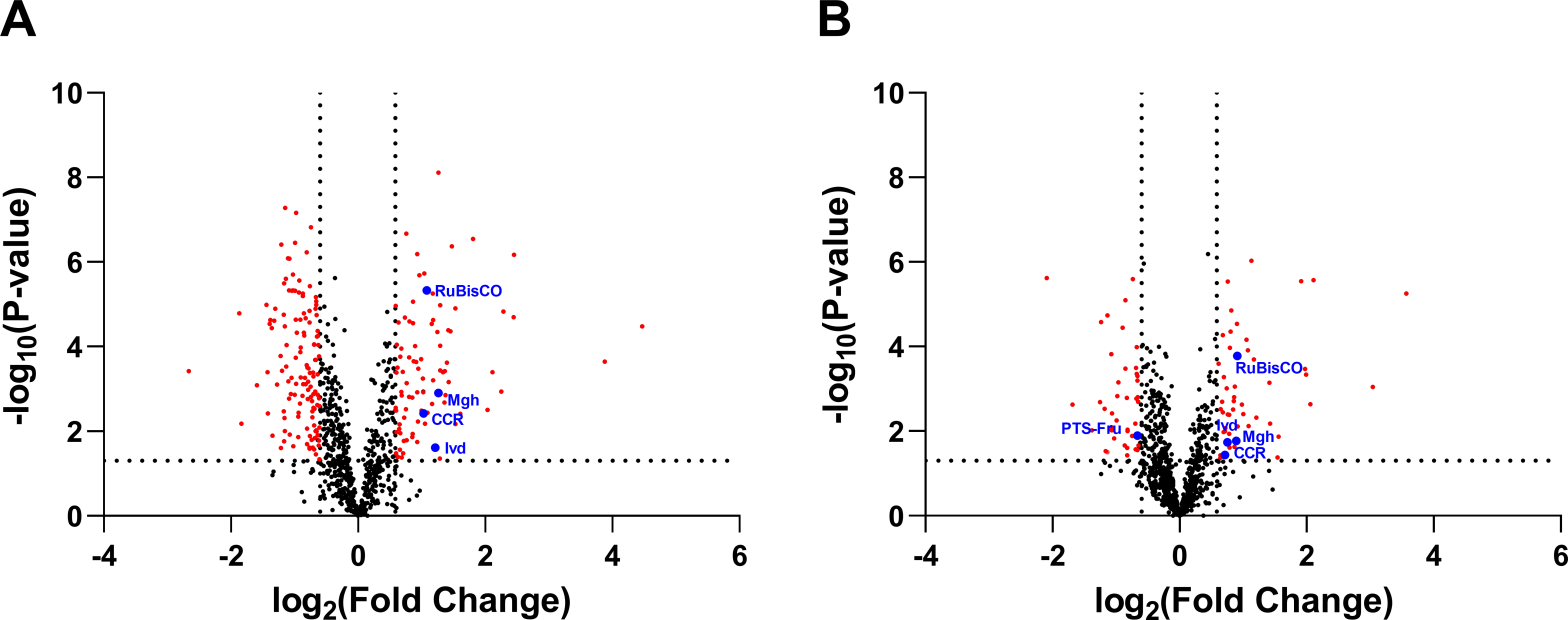
Volcano plots of the proteins quantified with significantly differential abundance between pure cultures of *Rs. rubrum* grown on previously autoclaved sucrose-containing medium and co-cultures *Rs. rubrum*/*Rh. capsulatus* grown in either previously filtered (**A**) or autoclaved (**B**) sucrose-containing culture medium. MS data were normalized at the species level to account for *Rs. rubrum* abundance in co-culture. The horizontal lines represent a *P-*value below 0.05, which is our significance threshold, and the vertical lines represent fold change values below 0.66 and above 1.5. RuBisco, ribulose-1,5-bisphosphate carboxylase/oxygenase; PTS-Fru, PTS fructose IIC component; CCR, crotonyl-CoA reductase; Ivd, isovaleryl-CoA dehydrogenase; and Mgh, methylglutaconyl-CoA hydratase.

For the analysis of *Rs. rubrum* in pure cultures vs co-cultures in autoclaved medium, 1,268 proteins were identified and quantified. Among them, 953 were detected with 2 or more peptides, of which 326 had a *P-*value less than or equal to 0.05. Of these 326 proteins, 51 were more abundant (fold change > 1.5) in co-cultures compared to pure cultures, while 45 were less abundant (fold change < 0.66). For the analysis of *Rs. rubrum* in pure cultures vs co-cultures in a filtered medium, 1,164 proteins were detected. Among them, 858 were identified with 2 or more peptides, of which 465 had a *P-*value of less than or equal to 0.05. Of these 465 proteins, 90 were more abundant (fold change > 1.5) in co-cultures compared to pure cultures, while 132 were less abundant (fold change < 0.66). Volcano plots showed that co-cultures in filtered medium had the greatest difference in abundance compared to pure cultures of *Rs. rubrum* (Fig. 5).

Fructose is transported by a PTS transport system and metabolized via the Embden-Meyerhof-Parnas pathway in *Rs. rubrum* (30). Our proteomic data revealed that one protein of the PTS system (PTS fructose IIC component, Rru_A1970) and one enzyme involved in fructose metabolism (PfkB, Rru_A1971) had a lower abundance in *Rs. rubrum* in co-culture with *Rh. capsulatus* than in *Rs. rubrum* in pure culture (Table 1). This suggests that *Rs. rubrum* assimilated less fructose in co-culture than in pure culture, regardless of the treatment applied to sucrose (filtered or autoclaved) and therefore might use another carbon source. We also showed that the ribulose-1,5-bisphosphate carboxylase/oxygenase (Rru_A2400, RuBisCO) exhibited a fold change of 1.87 (*P* < 0.001) and 2.12 (*P* < 0.001) in co-culture in previously autoclaved or filtered medium, respectively, compared to the pure culture. RuBisCO is known as the key enzyme of the Calvin-Benson-Bassham cycle, which is involved in CO_2_ fixation in PNSB. The presence of CO_2_ in the culture medium is required for the assimilation of substrates that are more reduced than biomass, such as butyrate, in order to regulate redox homeostasis in the cell by eliminating the reduced cofactors produced in excess (42, 43). It has also been shown that CO_2_ fixation is also required for the assimilation of substrates that are more oxidized than biomass, such as malate or succinate (43, 44). While these results could suggest that *Rs. rubrum* activates electron-sinking mechanisms to deal with a more reduced carbon source in co-culture with *Rh. capsulatus*, it is also possible that the observed increase in RuBisCO abundance is partially driven by differences in CO_2_ availability between pure and co-culture conditions. Indeed, previous work has shown that RubisCO expression in *Rs. rubrum* is sensitive to CO_2_ concentration, with transcriptional induction increasing at moderate CO_2_ levels and slightly decreasing at higher concentrations (45). Thus, the elevated RuBisCO levels in co-culture could result from redox balancing demands or from CO_2_-mediated transcriptional regulation.

**TABLE 1:** Differential protein expression in *Rs. rubrum* in co-culture with *Rh. capsulatus* in a sucrose-containing medium previously autoclaved or filtered^*a*^

UniProt accession no.	Locus tag	Co-culture in autoclaved medium/pure		Co-culture in filtered medium/pure		No. of identified peptides^*c*^	Description
		*P*-value	Protein fold change^*b*^	*P*-value	Protein fold change^*b*^		
Ethylmalonyl-CoA pathway							
Q2RPT6	Rru_A3064	1.83E−02	1.68	2.44E−02	2.32	15	Isovaleryl-CoA dehydrogenase
Q2RPT7	Rru_A3063	3.67E−02	1.64	3.81E−03	2.04	6	Crotonyl-CoA reductase
Q2RXX3	Rru_A0217	9.52E−01	1.00	5.35E−02	1.13	14	Citrate lyase
Q2RV43	Rru_A1201	1.46E−02	1.27	/^*d*^	/	9	MaoC-like dehydratase
Q2RPT8	Rru_A3062	2.12E−01	1.33	8.36E−02	1.49	2	Methylmalonyl-CoA mutase
Q2RU23	Rru_A1572	4.30E−01	1.14	8.77E−02	1.27	2	Methylmalonyl-CoA epimerase
Isoleucine production and degradation through methylbutyryl-CoA pathway							
Q2RT00	Rru_A1945	/	/	2.55E−05	1.75	6	Short-chain dehydrogenase/reductase SDR
Q2RVK4	Rru_A1040	2.56E−04	1.53	2.80E−05	1.82	3	Leucine dehydrogenase
Q2RX72	Rru_A0468	4.42E−05	1.75	2.34E−05	2.27	4	Acetolactate synthase, small subunit
Q2RX71	Rru_A0469	/	/	1.52E−07	0.60	11	Ketol-acid reductoisomerase
Q2RT17	Rru_A0470	1.01E−02	1.64	/	/	15	2-isopropylmalate synthase
Q2RXQ6	Rru_A0284	2.33E−03	1.97	6.65E−03	2.07	4	Acetolactate synthase, large subunit
Q2RS72	Rru_A2223	6.58E−05	1.47	3.86E−02	0.82	2	Branched chain amino acid: 2-keto-4-methylthiobutyrate aminotransferase/branched chain amino acid aminotransferase
Q2RX33	Rru_A0508	2.76E−03	1.35	5.40E−02	0.79	3	Aminotransferase, class IV
Q2RTB0	Rru_A1835	6.18E−03	2.55	4.25E−02	2.86	1	Butyryl-CoA dehydrogenase
Q2RXR6	Rru_A0274	1.10E−01	1.25	/	/	16	Acetyl-CoA acetyltransferase
Q2RTR3	Rru_A1682	2.87E−01	0.89	5.76E−01	0.90	10	Branched chain amino acid: 2-keto-4-methylthiobutyrate aminotransferase
Q2RT03	Rru_A1942	1.69E−02	1.86	1.24E−03	2.40	2	Methylglutaconyl-CoA hydratase
Q2RMQ0	Rru_A3801	5.61E−04	0.82	3.31E−04	0.67	13	Short-chain enoyl-CoA hydratase
Q2RTB0	Rru_A1835	6.18E−03	2.55	4.25E−02	2.86	1	Butyryl-CoA dehydrogenase
Calvin cycle							
Q2RRP5	Rru_A2400	1.65E−04	1.87	4.68E−06	2.12	22	Ribulose bisphosphate carboxylase
Fructose metabolism							
Q2RSX5	Rru_A1970	1.26E−02	0.63	5.34E−02	0.78	7	PTS fructose IIC component
Q2RSX4	Rru_A1971	1.08E−04	0.70	3.92E−03	0.79	9	PfkB

^*a*^ The complete data set can be found in Table S6.

^*b*^ The protein fold change is defined as the ratio of the abundance of a protein under two different conditions.

^*c*^ The number of identified peptides used for further analysis is with a higher confidence than 95%.

^*d*^ “/” indicates not detected.

Finally, differential proteomic analysis revealed that enzymes involved in the ethylmalonyl-CoA (EMC) pathway are more abundant in *Rs. rubrum* co-cultured with *Rh. capsulatus* than in pure cultures (Table 1). Among these, two key enzymes, isovaleryl-CoA dehydrogenase and crotonyl-CoA reductase, stood out with a fold change greater than 1.5 in the case of co-culture in previously autoclaved medium (FC = 1.68, *P* = 0.02 and FC = 1.64, *P* = 0.04, respectively) and greater than 2 in the case of co-culture in previously filtered medium (FC = 2.32, *P* = 0.02; and FC = 2.04, *P* = 0.004, for the isovaleryl-CoA dehydrogenase and the crotonyl-CoA reductase, respectively) compared to pure cultures. Moreover, our proteomic data also revealed that the isoleucine synthesis and degradation pathway (designated as methylbutanoyl-CoA or MBC pathway) appears to be upregulated in *Rs. rubrum* in co-cultures with *Rh. capsulatus* compared to pure cultures (Table 1). Previous studies carried out in our laboratory have shown that the EMC and MBC pathways are involved in the assimilation of acetate (8, 46), butyrate (47), and valerate (29), three volatile fatty acids. Taken together, these results are in favor of the hypothesis that *Rh. capsulatus* produces and excretes organic acids that *Rs. rubrum* can assimilate. It is important to emphasize that the observed upregulation of the majority of the key enzymes of the EMC and MBC pathways is greater for co-culture in filtered medium than in autoclaved medium (Table 1). This difference could reflect the fact that in autoclaved medium, part of the carbon source comes from fructose and glucose, whereas in filtered medium, only the organic acids produced and excreted by *Rh. capsulatus* could provide a carbon source for *Rs. rubrum.*

**Fig 6:**
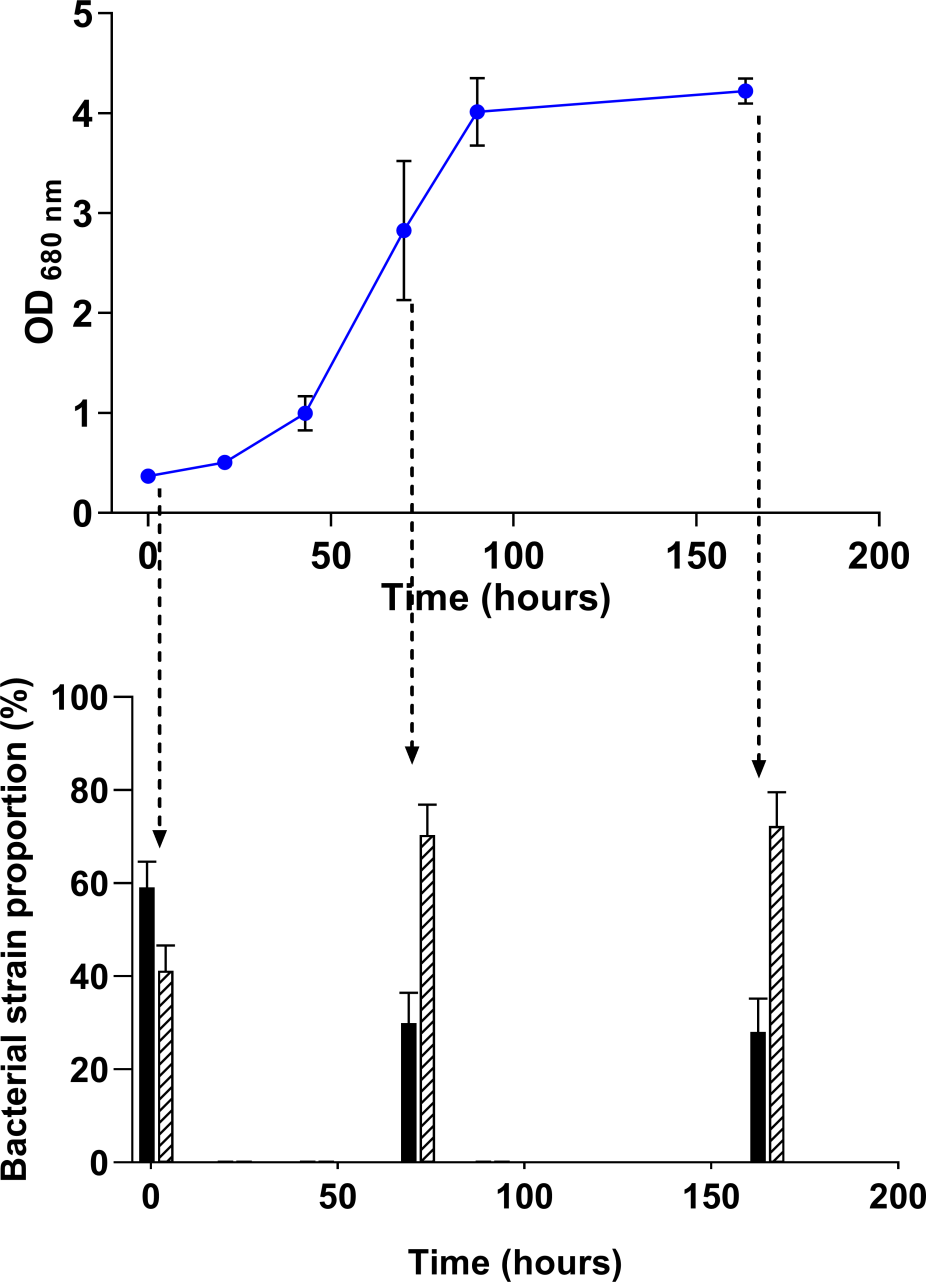
Growth monitoring of the co-culture of the *Δccr::*Kmr strain/*Rh. capsulatus* grown on previously filtered sucrose-containing medium and illuminated at 177 µmol photons m^−2^ s^−1^. Bar plots represent the proportion of the Δ*ccr::*Kmr strain (black bars) and *Rh. capsulatus* (hatched bars) at different time points. *n* = 5. Results are represented as the mean ± SD.

To further test this hypothesis and determine whether the EMC metabolic pathway is effectively required for the growth of *Rs. rubrum* in co-culture with *Rh. capsulatus*, we cultivated the *Rs. rubrum* Δ*ccr*::Kmr mutant strain (26) in co-culture with *Rh. capsulatus* with sucrose as the sole carbon source. This strain is mutated in the gene coding for crotonyl-CoA carboxylase/reductase (Rru_A3063), a key enzyme of the EMC metabolic pathway, thereby making it deficient in acetate assimilation. Using proteomic analysis, we investigated the proportions of *Rs. rubrum* and *Rh. capsulatus* at three time points of the growth phase*.* Interestingly, a decrease in the relative abundance in the co-culture was observed with the Δ*ccr*::Kmr mutant strain, representing 27.9% ± 7.4% of the bacterial population (Fig. 6), whereas under the same culture conditions, the WT strain represented 38.1% ± 4.8% of the co-culture at the end of the growth (Fig. 4A). Notably, although the Δ*ccr*::Kmr strain started the culture with a higher initial proportion (58.9% ± 5.6%) than the WT strain (46.3% ± 2.3%), it nevertheless reached a lower final proportion. This trend may suggest a reduced division rate of the Δ*ccr*::Kmr strain in co-culture; however, additional experiments would be required to confirm this hypothesis. Taken together, these results suggest that while the EMC pathway is not strictly essential for *Rs. rubrum* growth in co-culture with *Rh. capsulatus*, the Δ*ccr*::Kmr mutant tends to reach a lower final proportion in the co-culture than the WT strain. In previous studies, we showed that the EMC pathway is only essential for acetate assimilation (26), but not for butyrate or valerate assimilation (29). We thus hypothesized that, in our co-culture conditions, *Rs. rubrum* partially relied on acetate as a carbon source but probably also used other molecules released by *Rh. capsulatus,* such as lactic acid and formic acid.

This hypothesis was further supported by the analysis of the culture medium composition during the exponential phase, which revealed no detectable accumulation of organic acids or only very low amounts (~1 mM in the case of formate). Consistently, as already mentioned, organic acid assimilation by *Rs. rubrum* limits the pH drop between 0.5 and 0.8 units (Fig. S6), which is much less than the decrease observed when *Rh. capsulatus* was cultivated alone and which was responsible for incomplete sugar assimilation in the absence of thiamine supplementation (Fig. 2A). The association of these two PNSB allows more efficient sucrose utilization (0.64 g dry weight L⁻¹ per g sucrose L⁻¹) and valorization compared to pure cultures (0.12 and 0.29 for *Rs. rubrum* and *Rh. capsulatus*, respectively), relieving the need for preliminary sucrose hydrolysis required for *Rs. rubrum* or the supplementation with thiamine, required for *Rh. capsulatus*. It is important to note that a transfer of thiamine from *Rs. rubrum,* which can synthesize it, to *Rh. capsulatus*, which requires thiamine for photoheterotrophic sugar assimilation, may also occur. This hypothesis requires further investigation.

**Fig 7:**
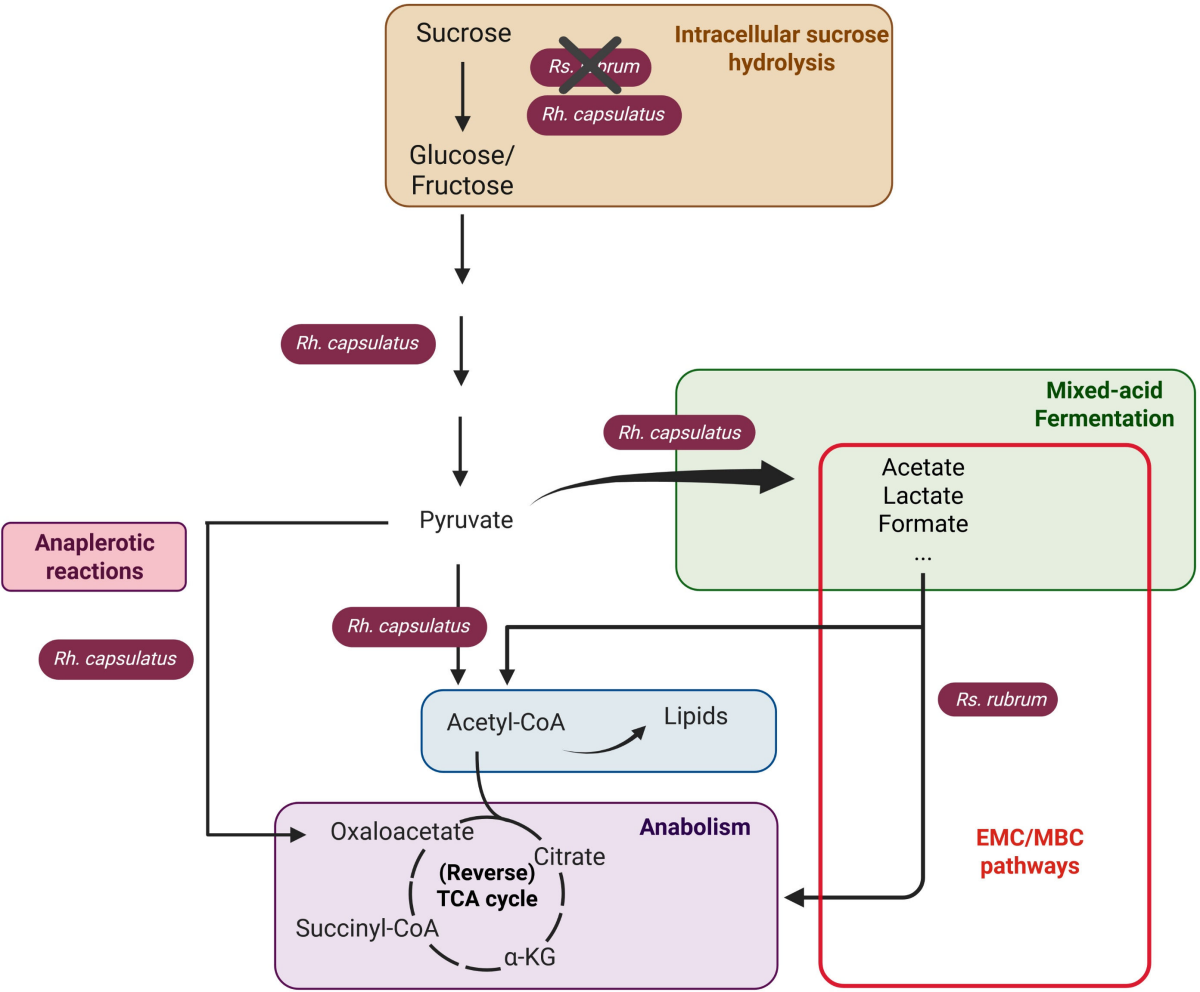
Schematic representation of the proposed metabolic interaction between *Rs. rubrum* and *Rh. capsulatus* in photoheterotrophy under anaerobic conditions and without thiamine supplementation, using sucrose as the sole carbon source. *Rh. capsulatus* hydrolyzes sucrose into glucose and fructose, whereas *Rs. rubrum* lacks this ability. In *Rh. capsulatus,* these carbohydrates are processed through glycolysis, generating pyruvate, which is used in anabolic metabolism and partly converted into organic acids, such as lactate, formate, and acetate via mixed-acid fermentation. These fermentation products are subsequently assimilated by *Rs. rubrum* through the EMC/MBC pathways. This figure illustrates a trophic interaction in which *Rh. capsulatus* supports *Rs. rubrum* growth through the release of organic acids, while *Rs. rubrum* contributes to maintaining pH stability, enabling *Rh. capsulatus* phototrophic growth. Created in BioRender (M. Gilson, 2025).

The co-culture thus revealed a trophic interaction between *Rs. rubrum* and *Rh. capsulatus* in the presence of sucrose and in the absence of thiamine. *Rh. capsulatus* intracellularly hydrolyzes sucrose into glucose and fructose and ferments these sugars into organic acids, which are subsequently assimilated by *Rs. rubrum*. These metabolites are then assimilated by *Rs. rubrum*, supporting its growth. This process supports the growth of *Rs. rubrum* and, in turn, promotes *Rh. capsulatus* growth by preventing a drop in the medium’s pH (Fig. 7).

### Assimilation of sucrose under photobioreactor conditions

**Fig 8:**
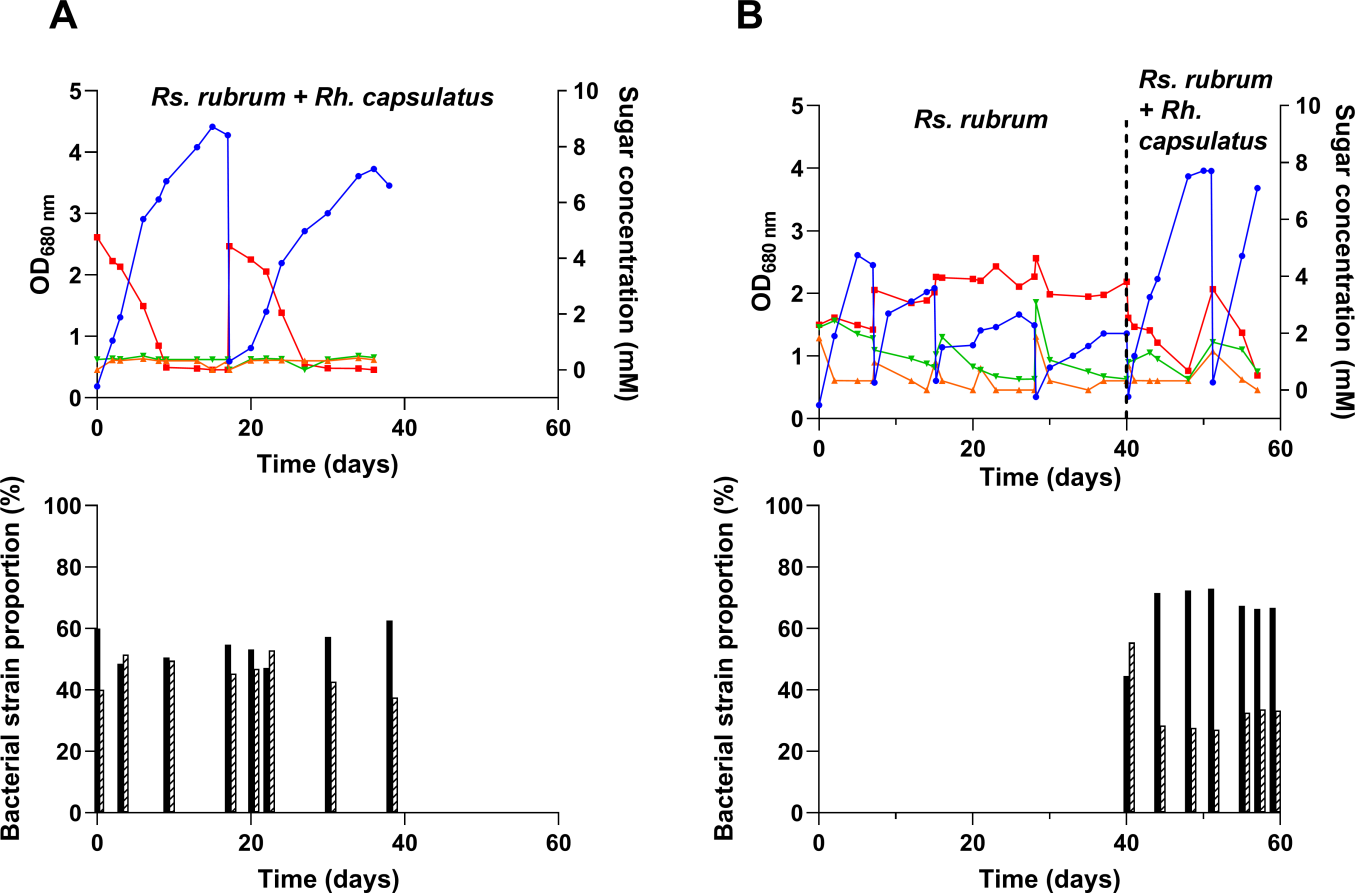
Monitoring of growth (blue line) of co-cultures *Rs. rubrum*/*Rh. capsulatus* grown on either previously filtered (**A**) or autoclaved (**B**) sucrose-containing medium under sequential batch mode and illuminated at ±400 µmol photons m⁻² s⁻¹. In the latter condition (**B**), the culture was initially started with *Rs. rubrum* alone, and *Rh. capsulatus* was added after 40 days of cultivation. Bar plots represent the proportion of *Rs. rubrum* (black bars) and *Rh. capsulatus* (hatched bars) at different time points. Red, green, and orange lines represent the evolution of sucrose, glucose, and fructose concentration, respectively. *n* = 1.

A trophic link might be very promising for the development of PNSB-based resource recovery strategies. To determine if the flask-identified trophic link between *Rs. rubrum* and *Rh. capsulatus* also applies at a larger scale, autoclaved (hydrolyzed) or filtered (non-hydrolyzed) sucrose was used as a sole carbon source for a co-culture of these strains under photobioreactor conditions. Bacterial growth has been observed in both filtered (two sequential batches) and autoclaved medium (six sequential batches) (Fig. 8). Consistent with the observations from the flask experiment, total carbon assimilation was observed on filtered medium in the presence of a co-culture of *Rs. rubrum* and *Rh. capsulatus* (Fig. 8A), reaching an OD_680_ comparable to those observed during the flask experiments with an average productivity of 0.16 ± 0.05 g/L·day, which is in line with already published data using a sequential batch reactor (18). Proteomic analysis was used to evaluate the proportions of both strains and revealed that, as in flask experiments, *Rs. rubrum* was growing in a non-hydrolyzed sucrose medium in PBR (Fig. 8A). Unexpectedly, *Rs. rubrum* even dominated the co-culture at certain time points, suggesting an effect of the PBR conditions in improving its fitness in addition to the synergistic effect of the presence of *Rh. capsulatus*. This observation further demonstrates that upscaling can alter the production process and thus the key parameters (e.g., productivity, carbon yield, growth rate, and COD removal) of the process. The dominance of *Rs. rubrum* in the PBR could be due to more appropriate illumination in this setup (400 µmol photons m⁻² s⁻¹ vs 177 µmol photons m⁻² s⁻¹ in flask experiments) or to the difference in mixing strategy (orbital shaking for flask experiments vs internal Rushton mixing for PBR).

Moreover, in flask experiments, we have shown that *Rs. rubrum* relied more on phototrophy than *Rh. capsulatus*, as indicated by the higher amounts of photosynthetic pigments (Fig. 2C and D). It is therefore possible that the more uniform light penetration in the PBR further supports *Rs. rubrum* growth compared to *Rh. capsulatus* growth.

We further wanted to determine if the trophic interaction also occurs in PBR when partially hydrolyzed sucrose was used as the carbon source. We performed four sequential batches on autoclaved sucrose-containing medium with *Rs. rubrum* only (Fig. 8B, days 0–40). Reflecting data obtained in flasks, the growth was limited, reaching an OD_680_ of ~2.0 (Fig. 8B). As shown by the monitoring of carbon source assimilation, this growth corresponds to the assimilation of fructose and glucose derived from the hydrolysis of sucrose during autoclaving, further demonstrating the absence of sucrose assimilation by *Rs. rubrum*. As in flask experiments, productivity was significantly higher during the assimilation of fructose (0.3 ± 0.06 g/L·day) than during the assimilation of glucose (0.03 ± 0.004 g/L·day). After these four sequential batches with *Rs. rubrum* only, we added *Rh. capsulatus* to the culture. The addition of *Rh. capsulatus* triggered the full consumption of the carbohydrates in the medium and a significant increase in the maximal OD_680_ reached in the photobioreactor. Moreover, the observed productivity of the co-culture also rose to reach 0.33 ± 0.05 g/L·day, which corresponds to the one observed for *Rs. rubrum* during fructose assimilation (0.27 ± 0.06 g/L·day) and is comparable to previously reported values for sequential batch mode (18) (*t*-test, *P*-value < 0.05). The use of hydrolyzed sucrose resulted in a large dominance of *Rs. rubrum* over *Rh. capsulatus,* as the former strain reached up to almost 80% of the co-culture. This dominance of *Rs. rubrum* in the hydrolyzed sucrose condition was not observed in flasks but reflects the trend observed in PBR with filtered sucrose-containing medium. *Rs. rubrum* also underwent four successive sequential batches in the PBR before the addition of *Rh. capsulatus,* which might have led to the emergence of a better-adapted strain, consistent with the strong adaptation capacity of this strain (26, 27).

### Conclusion

In this study, we investigated the differences in sucrose assimilation capacity of two phototrophic purple non-sulfur bacteria, as sucrose-containing by-products represent an important feedstock for the development of biotechnological resource recovery and upcycling strategies. Although culture media containing sucrose in the form of molasses have been largely studied as carbon sources for PNSB, we revealed that sucrose cannot be assimilated by *Rs. rubrum* unless it is previously hydrolyzed. *Rh. capsulatus* could use non-hydrolyzed sucrose but seems to rely on a mixed-acid fermentation pathway instead of photoheterotrophic growth, thereby leading to both a lower carbon yield and a lower nutrient recovery. We observed that thiamine supplementation in *Rh. capsulatus* cultures improved the fructose assimilation capacity by reducing the shift toward a fermentation metabolism, thus probably preventing the accumulation of intracellular pyruvate. Setting up a co-culture of these two strains significantly improved the carbon utilization in both hydrolyzed and non-hydrolyzed sucrose-containing medium without the need for thiamine supplementation. *Rs. rubrum* became capable of thriving in non-hydrolyzed sucrose conditions when co-cultivated with *Rh. capsulatus*. We could determine that a trophic link is established between the two strains when co-cultivated, with *Rs. rubrum* growing photoheterotrophically on the fermentation products of *Rh. capsulatus*. The co-cultivation not only increased the carbon yield but also the productivity and could probably be further used to tune co-culture composition, notably for dedicated biomass quality production. Finally, this synergistic effect could also be observed in the PBR condition, highlighting the potential for developing enhanced resource recovery strategies based on microbial metabolic interactions and demonstrating how microbial co-cultures can improve the valorization of organic substrates, making these findings particularly relevant for the circular economy.

## Data Availability

Proteomic data sets, files, and parameters are freely available in the online MassIVE repository with the identifier MSV000096927. The full and detailed protocols of sample preparation for mass spectrometry analysis have been submitted to protocols.io and are available at the following link: https://www.protocols.io/private/44AF9D46D73D11EF80010A58A9FEAC02. The full and detailed protocols of organic acid derivatization, detection, and quantification using mass spectrometry have been submitted to protocols.io and are available at the following link: https://www.protocols.io/private/C8996654F04511EF81B10A58A9FEAC02.

## SUPPLEMENTAL MATERIAL

Data S1 mbio.03717-25-s0001.pdf Implementation of mass spectrometry-based proteomic analysis to estimate strain proportions in PNSB co-cultures, as an alternative to CFU counts and 16S rRNA sequencing.

Supplemental Figures mbio.03717-25-s0002.docx Fig. S1 to S6.

Supplemental Note mbio.03717-25-s0003.pdf Description of medium compositions and sterilization methods.

Table S1 mbio.03717-25-s0004.xlsx Emission spectrum of halogen lamps.

Table S2 mbio.03717-25-s0005.xlsx Calibration curve of dry cell weight in function of OD680 used to determine biomass concentration for *Rs. rubrum* and *Rh. capsulatus*.

Table S3 mbio.03717-25-s0006.xlsx Summary of growth yields, biomass productions, and productivities for all experimental conditions.

Table S4 mbio.03717-25-s0007.xlsx Complete data set of proteomic analysis comparing the proteome of *Rs. rubrum* during fructose uptake with its proteome during glucose uptake.

Table S5 mbio.03717-25-s0008.xlsx Organic acid concentrations measured under the different growth conditions for *Rh. capsulatus*, *Rs. rubrum*, and the co-culture.

Table S6 mbio.03717-25-s0009.xlsx Complete data set of proteomic analysis comparing the proteome of *Rs. rubrum* in pure cultures with its proteome in co-cultures with *Rh. capsulatus*.

ASM does not own the copyrights to Supplemental Material that may be linked to, or accessed through, an article. The authors have granted ASM a non-exclusive, world-wide license to publish the Supplemental Material files. Please contact the corresponding author directly for reuse.
