# Supplementary material for: Fundamental aspects of sucrose metabolism reveal a trophic link between Rhodospirillum rubrum and Rhodobacter capsulatus
Source: mBio. 2026 Feb 13;17(3):e03717-25. doi: 10.1128/mbio.03717-25 (PMC12977620; doi:10.1128/mbio.03717-25)
Supplement: Data S1 — Implementation of mass spectrometry-based proteomic analysis to estimate strain proportions in PNSB co-cultures, as an alternative to CFU counts and 16S rRNA sequencing. [file mbio.03717-25-s0001.pdf]

Supplementary Data 1

Legend: To estimate strain proportions in PNSB co-cultures, mass spectrometry-based proteomics was tested as an alternative to CFU counts and 16S rRNA sequencing, which are affected by growth differences and PCR biases, respectively.

To determine strain proportions in a culture, most studies rely on counting colony-forming units (CFU) or more recently on 16S rRNA gene amplicon sequencing. These methods are prone to important bias due to differences in growth capacity on solid medium among different strains or to differences in the PCR amplification efficiency when relying on degenerated primers. In order to better follow composition in co-culture of PNSB we decided to test the use of proteomic analysis by mass spectrometry (MS) as a proxy of strain abundance.

Five precultures of three strains of purple bacteria (*Rhodobacter capsulatus* ATCC 11166, *Rhodospirillum rubrum* S1H ATCC 25903 and *Cereibacter sphaeroides* ATCC 17029) were grown in SMN (supplemented malate-ammonium) medium until stationary phase. The OD_{680} was adjusted to around 0.133 for each strain (final OD_{680} ~ 0.4), before mixing them in equal parts to set up five replicates of a tripartite co-culture in SMN medium. Composition of the co-culture was determine using the three methods, to test their capacity to retrieve the even composition of the tripartite culture. To determine the CFU/mL, samples were diluted by serial 10-fold dilutions and 100 µL of the respective dilutions were spread on SMN agar plates. The agar plates were incubated for 2-5 days and the appearing colonies were counted daily. One ml of the cultures were centrifuged at 5000 × *g* for 15 min to recover the pellets to perform the 16S rRNA gene sequencing and the proteomic analysis. The DNA extraction was performed using the DNeasy® PowerSoil® kit (Qiagen, 47014) and the DNA concentration was determined using the QuantiFluor® ONE dsDNA system (Promega, E4870) and Quantus™ Fluorometer (Promega, E6150). The sequencing library was prepared using the 16S Barcoding Kit 24 V14 (Oxford Nanopore, SQK-16S114.24) and sequenced using MinION GridION Flow cells R9 version (Oxford Nanopore, FLO-MIN106D.8) on a GridION Mk1 device (Oxford Nanopore, GRD-X5B003). To analyse the sequencing data, an EPI2ME workflow was used to assign the genera and species names, and the ratio between the number of reads and total number of reads was calculated. The Data dependent acquisition (DDA) of the mass spectrometry was used as described in the section "strain quantification" of the Material and Methods of this article. The combined proteomes of *Rh. capsulatus, Rs. rubrum* and *Ce. sphaeroides* from UniProt were used as a database and a false discovery rate (FDR) < 1% was applied, which allowed to count the number of proteins of each species. The proportion of a strains was calculated as the ration of the number of proteins belonging to this species (unshared peptides only) on the total number of identified proteins. As the co-cultures were set up to contain one third of each strain, it was expected that the results would reflect these proportions. However, the results showed that the proteomic analysis by mass spectrometry most closely matched the composition of the culture. The CFU counting was reliable for the counting of colonies from *Rs. rubrum* due to their later appearance on the agar plates and smaller colony size. However, colonies from *Rh. capsulatus* and *Ce. sphaeroides* had a similar morphology, which hampered the confident identification of these colonies. In addition, the co-culture seemed to have an effect on the moment of appearance of those colonies compared to pure cultures. The 16S rRNA gene sequencing revealed a preferential PCR amplification of the 16S of *Rh. capsulatus*. All strains contained 4 copies of the 16S rRNA gene (https://rrndb.umms.med.umich.edu/genomes/taxonomy/, Accessed 08/10/2024). An additional experiment where the same quantity of DNA was added to the amplification revealed the same preferential PCR amplification. These results show that the PCR amplification could be a major

hindrance in the correct identification of bacterial strain proportions. Future experiments could focus on using different primers to reduce this bias. The proteomic analysis using mass spectrometry revealed the bacterial strain proportions we were expecting of around 33% of each. Mass spectrometry analysis for strain proportions could therefore become a powerful tool in the future.

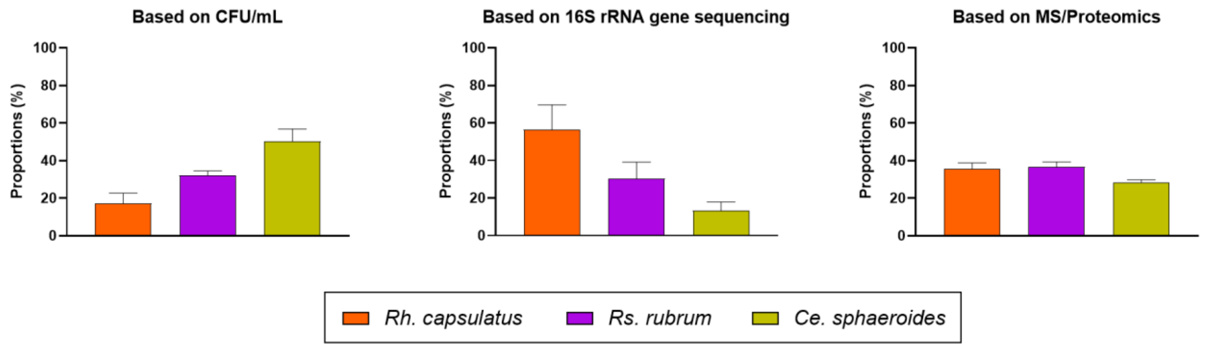

The bacterial strain proportions of *Rh. capsulatus, Rs. rubrum* and *Ce. sphaeroides* which were inoculated at an OD_{680} of around 0.133 each. The proportions were determined using colony-forming unit counting (CFU/mL), 16S rRNA gene sequencing and proteomic analyses using mass spectrometry. The latter revealed a similar proportion of each strain. (Mean ± SD, n = 5 biological replicates).
