# Supplementary material for: Fundamental aspects of sucrose metabolism reveal a trophic link between Rhodospirillum rubrum and Rhodobacter capsulatus
Source: mBio. 2026 Feb 13;17(3):e03717-25. doi: 10.1128/mbio.03717-25 (PMC12977620; doi:10.1128/mbio.03717-25)
Supplement: Supplemental Figures — Fig. S1 to S6. [file mbio.03717-25-s0002.docx]

**
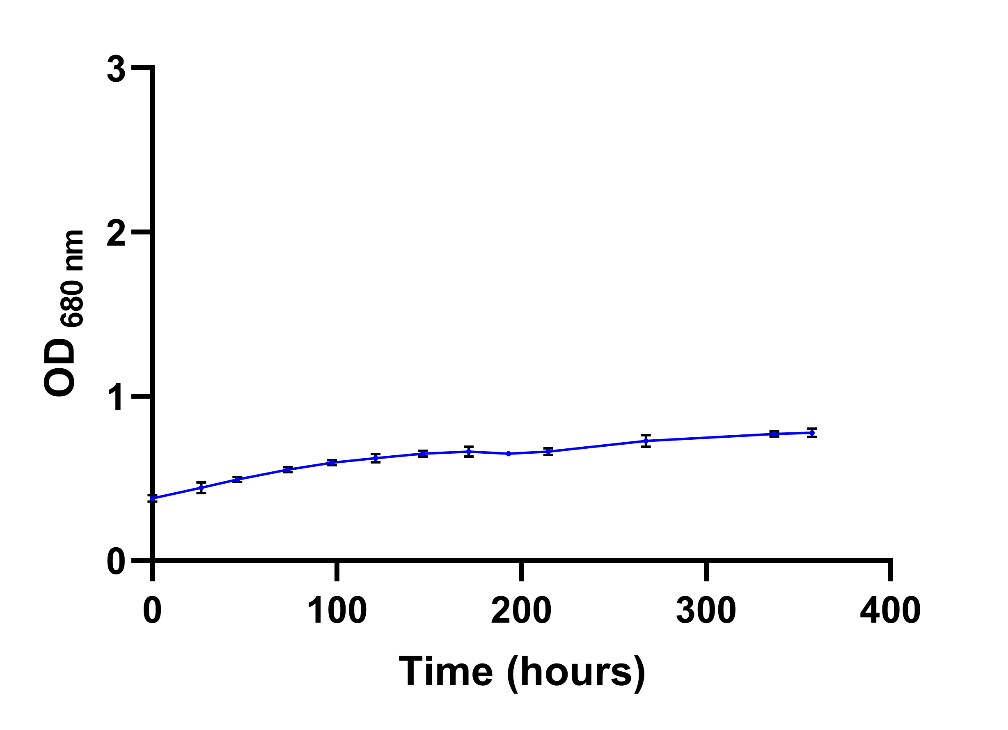
Supplementary Figures**

**Supplementary Figure 1 |** Monitoring of growth of *Rhodospirillum rubrum* cultivated in a medium containing 20 mM glucose as carbon source and illuminated at 177 µmol photons m⁻² s⁻¹. *n* = 5. Results are represented as the mean ± SD.

**
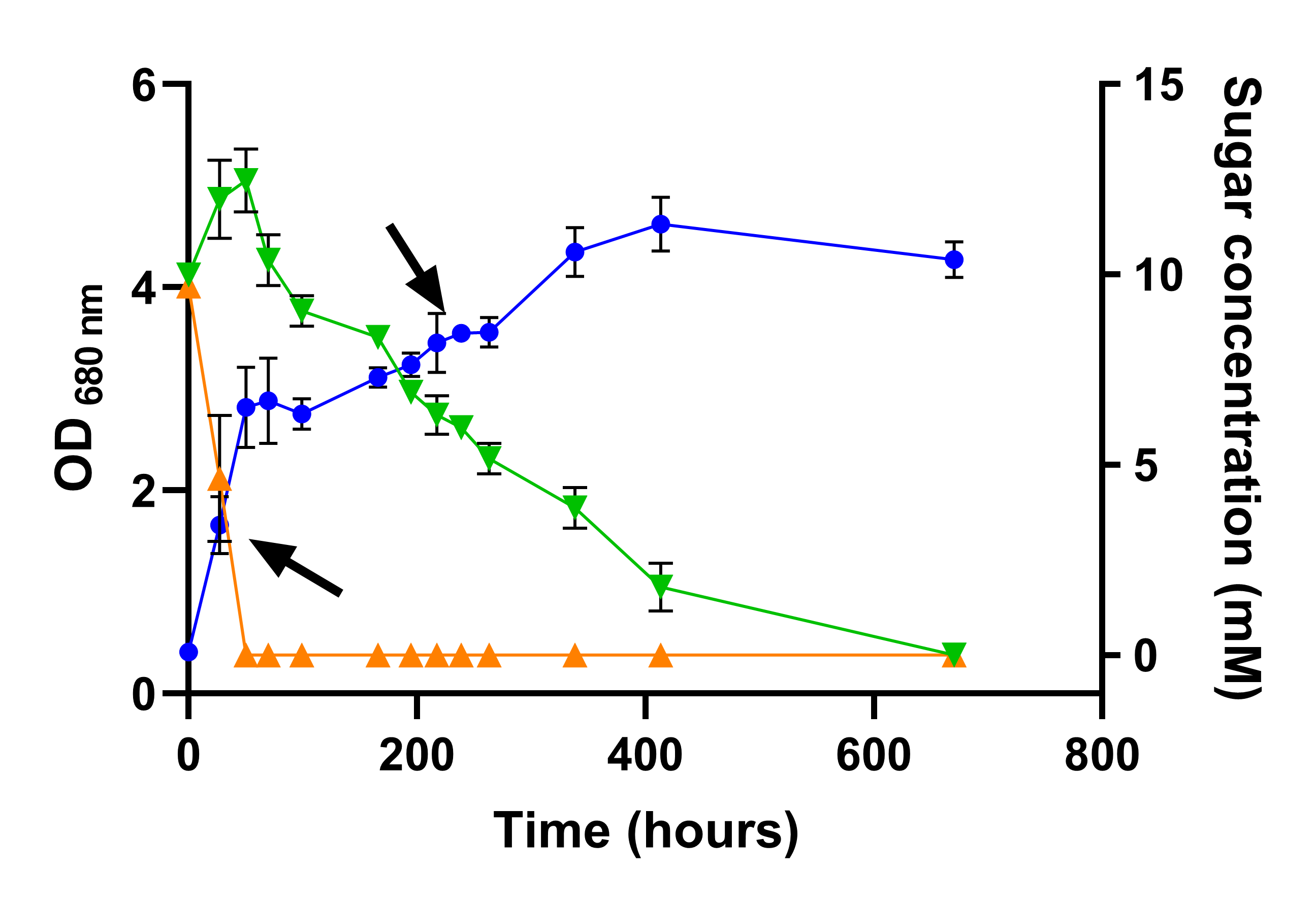
**

**Supplementary Figure 2 |** Monitoring of growth (blue line) of *Rhodospirillum rubrum* cultivated in a medium containing fructose and glucose (50/50, 120 mM carbon equivalent) as carbon source and illuminated at 177 µmol photons m⁻² s⁻¹. Green and orange lines represent the evolution glucose and fructose concentration, respectively. Sampling times for proteomic analyses are indicated by black arrows. *n* = 5. Results are represented as the mean ± SD.


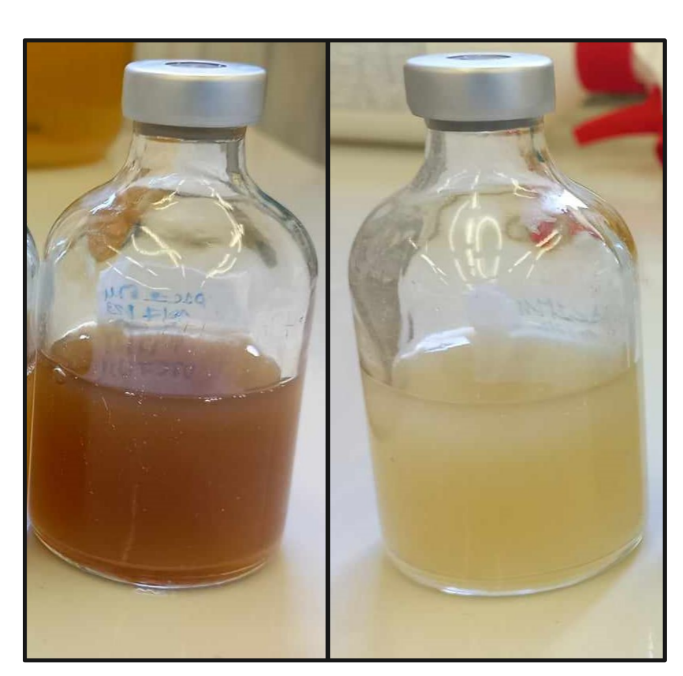


**Supplementary Figure 3 |** Final culture colours of Rh. capsulatus grown in medium containing succinate (left) or fructose (right) as carbon sources and without thiamine supplementation.


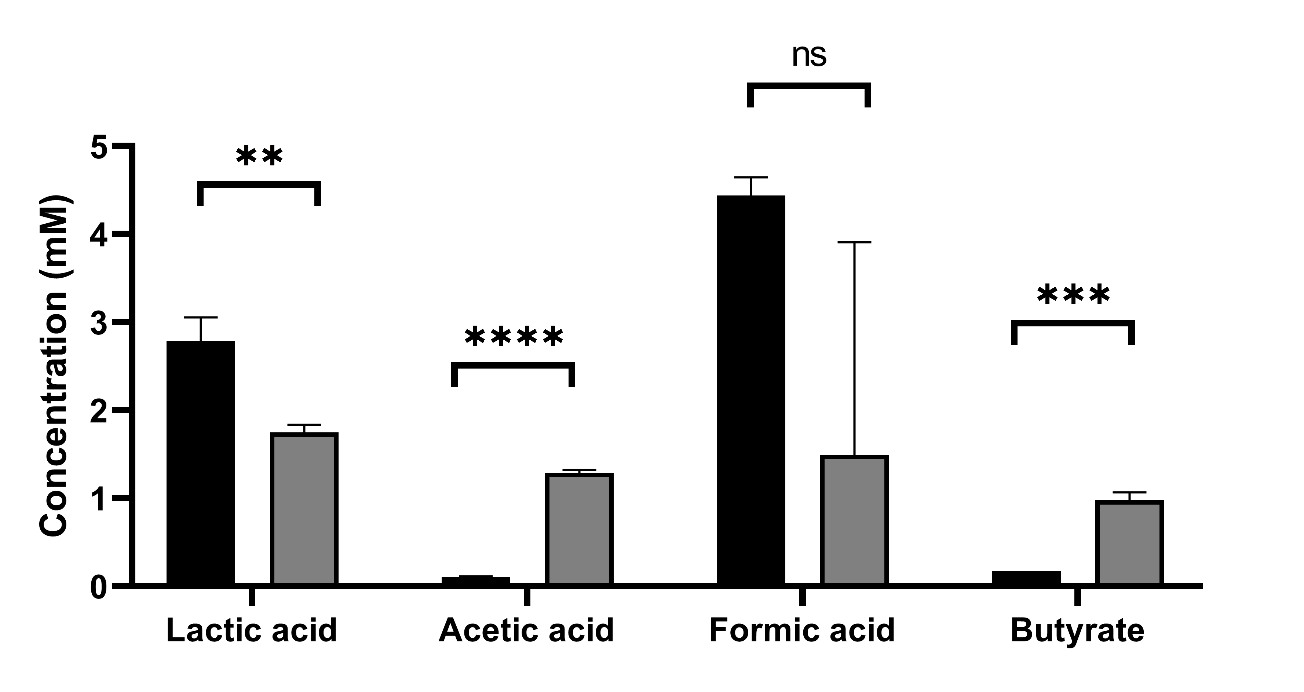


**Supplementary Figure 4 |** Determination and quantification of the organic acids produced by Rh. capsulatus cultivated in a 20 mM fructose-containing medium without (black bars) or with (grey bars) thiamine supplementation (n = 3; **: p value<0.005; ***: p value<0.0005; ****: p value<0.0001). Results are represented as the mean ± SD.


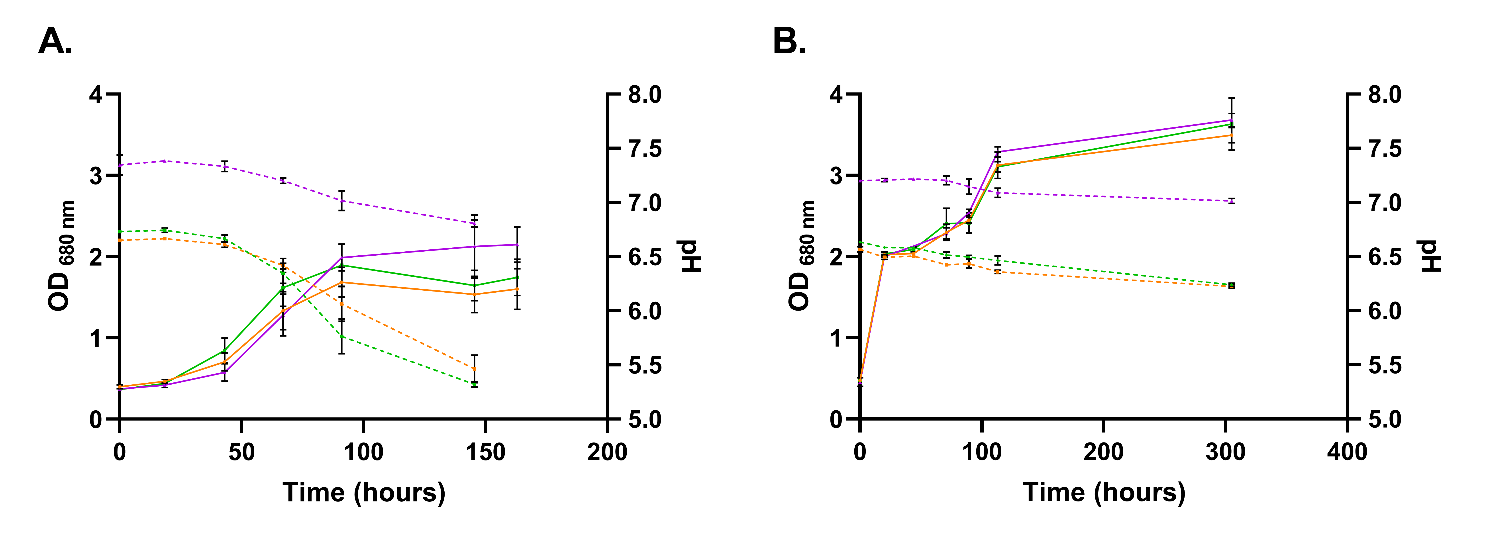


**Supplementary Figure 5 |** Monitoring of growth (solid lines) and pH variation (dotted lines) of Rh. capsulatus **(A)** and Rs. rubrum **(B)** cultivated in a medium containing autoclaved sucrose (partially hydrolysed) under illumination at 177 µmol photons m^-2^ s^-1^. Cultures were supplemented with bicarbonate ions at 3 mM (green lines), 50 mM (purple lines), or grown without supplementation (orange lines). n = 3. Results are represented as the mean ± SD.


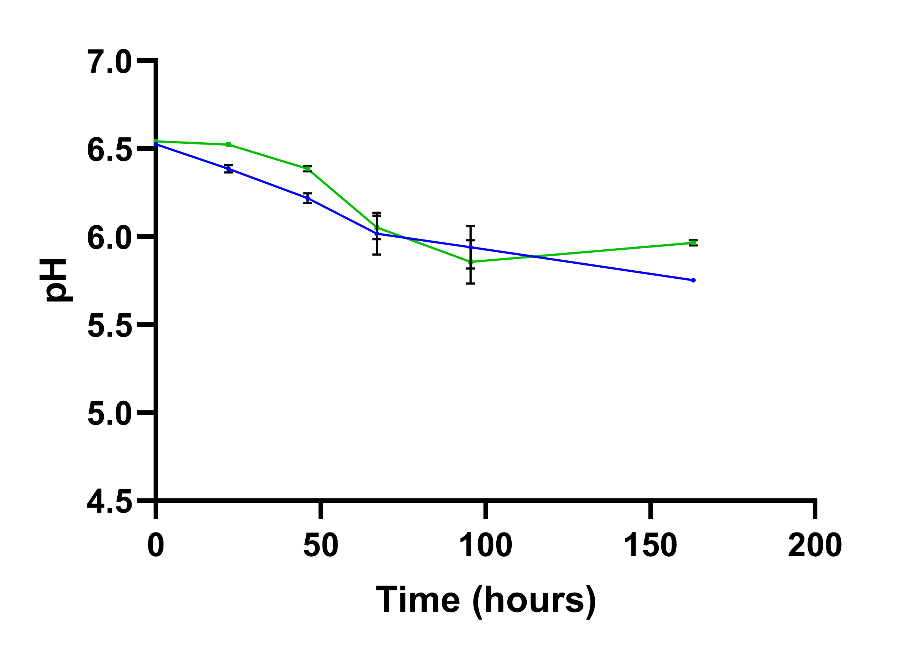


**Supplementary Figure 6 |** pH evolution of co-cultures Rs. rubrum/Rh. capsulatus grown on either previously filtered (green line) or autoclaved (blue line) sucrose-containing medium and illuminated at 177 µmol photons m^-2^ s^-1^. n = 3. Data represent the mean ± SD.
