# Supplementary material for: Fundamental aspects of sucrose metabolism reveal a trophic link between Rhodospirillum rubrum and Rhodobacter capsulatus
Source: mBio. 2026 Feb 13;17(3):e03717-25. doi: 10.1128/mbio.03717-25 (PMC12977620; doi:10.1128/mbio.03717-25)
Supplement: Supplemental Note — Description of medium compositions and sterilization methods. [file mbio.03717-25-s0003.pdf]

Legend: This document details the composition of the media used in the study, as well as the sterilisation procedures.

Recipe and procedure of sterilisation

Sterilisation procedure – autoclaving:

Autoclave model: TouchClave-R 120L Vertical from LTE

Mode: Ballast

Step 1: Water fill

Step 2: Pre-vacuum → decreases to 500 mbar during 1 minute.

Step 3: Steam Purge → 90°C for 5 minutes

Step 4: Heat up.

Step 5: Sterilisation → 121°C and 2150 mbar for 30 minutes

Step 6: Cooling → 80°C for 5 minutes

Sterilisation procedure – filtration:

Sterilisation through a filtration membrane with pore size of 0.2 µm (ref.: Thermo Scientific™ 290-4520)

Medium composition:

Components	Concentration (mM)
$MnCL_2.4H_2O$	0.05
$MgSO_4.7H_2O$	0.8
Na_2SO_4	3.8
NH_4Cl	35.3
$CaCl_2.H_2O$	0.34
Sucrose/Fructose/Succinate	10/20/30 (120 mM C eq.)
EDTA	0.07
KH_2PO_4	3.6
$FeSO_4.7H_2O$	0.07
MOPS	100.3
K_2HPO_4	2.98
$NiSO_4.6H_2O$	0.0019
$ZnSO_4.7H_2O$	0.00035
$CuSO_4.5H_2O$	0.00002
H_3BO_3	0.0016
$Na2MoO_4.2H_2O$	0.0002
Biotin	0.00006

<u>SMN medium composition</u>

Components	Concentration (mM)
EDTA	0.07
KH_2PO_4	4.4
Malate	44.7
$FeSO_4$•$7H_2O$	35.3
$MgSO_4$•$7H_2O$	0.34
$(NH_4)_2SO_4$	10
K_2HPO_4	0.07
$CaCl_2$•$2H_2O$	3.6
Yeast Extract	0.07
Peptone	100.3
$ZnSO_4.7H_2O$	0.0008
$Cu(NO_3)$•$3H_2O$	0.0002
H_3BO_3	0.045
$MnSO_4$•$4H_2O$	0.0094
$MoNa_2O_4$•$2H_2O$	0.0017
Biotin	0.00006
